# The human pathobiont *Malassezia furfur* secreted protease Mfsap1 regulates cell dispersal and exacerbates skin inflammation

**DOI:** 10.1073/pnas.2212533119

**Published:** 2022-11-29

**Authors:** Joleen P. Z. Goh, Fiorella Ruchti, Si En Poh, Winston L. C. Koh, Kiat Yi Tan, Yan Ting Lim, Steven T. G. Thng, Radoslaw M. Sobota, Shawn S. Hoon, Chenxi Liu, Anthony J. O’Donoghue, Salomé LeibundGut-Landmann, Hazel H. Oon, Hao Li, Thomas L. Dawson

**Affiliations:** ^a^A*STAR Skin Research Labs, Agency for Science, Technology and Research, Singapore 138648; ^b^Lee Kong Chian School of Medicine, Nanyang Technological University Singapore, Singapore 308232; ^c^Section of Immunology, Vetsuisse Faculty and Institute of Experimental Immunology, University of Zürich, Zürich CH-8057, Switzerland; ^d^Molecular Engineering Lab, Institute of Molecular and Cell Biology, Agency for Science, Technology and Research, Singapore 138673; ^e^Institute of Bioengineering and Bioimaging, Agency for Science, Technology and Research, Singapore 138669; ^f^Bioinformatics Institute, Agency for Science, Technology and Research, Singapore 138671; ^g^Functional Proteomics Laboratory, Institute of Molecular and Cell Biology, Agency for Science, Technology and Research, Singapore 138673; ^h^National Skin Centre, National Healthcare Group, Singapore 308205; ^i^Skin Research Institute of Singapore, Agency for Science, Technology and Research, Singapore 308232; ^j^Skaggs School of Pharmacy and Pharmaceutical Sciences, University of California San Diego, La Jolla, CA 92093-0657; ^k^Department of Chemistry, National University of Singapore, Singapore 117543; ^l^Department of Drug Discovery, School of Pharmacy, Medical University of South Carolina, Charleston, SC 29425

**Keywords:** skin, fungi, protease, colonization, inflammation

## Abstract

*Malassezia* form the dominant eukaryotic microbial community on the human skin. The *Malassezia* genus possesses a repertoire of secretory hydrolytic enzymes involved in protein and lipid metabolism which alter the external cutaneous environment. The exact role of most *Malassezia* secreted enzymes, including those in interaction with the epithelial surface, is not well characterized. In this study, we compared the expression level of secreted proteases, lipases, phospholipases, and sphingomyelinases of *Malassezia globosa* in healthy subjects and seborrheic dermatitis or atopic dermatitis patients. We observed upregulated gene expression of the previously characterized secretory aspartyl protease *MGSAP1* in both diseased groups, in lesional and non-lesional skin sites, as compared to healthy subjects. To explore the functional roles of *MGSAP1* in skin disease, we generated a knockout mutant of the homologous protease *MFSAP1* in the genetically tractable *Malassezia furfur*. We observed the loss of *MFSAP1* resulted in dramatic changes in the cell adhesion and dispersal in both culture and a human 3D reconstituted epidermis model. In a murine model of *Malassezia* colonization, we further demonstrated Mfsap1 contributes to inflammation as observed by reduced edema and inflammatory cell infiltration with the knockout mutant versus wildtype. Taken together, we show that this dominant secretory *Malassezia* aspartyl protease has an important role in enabling a planktonic cellular state that can potentially aid in colonization and additionally as a virulence factor in barrier-compromised skin, further highlighting the importance of considering the contextual relevance when evaluating the functions of secreted microbial enzymes.

## Significance

Our skin is the primary exposure site of external microbiota, including the dominant fungus, *Malassezia*. The continual exchange of communication molecules between the microbes and the host enables host immune system sensing and response to the microbiota and vice versa. Here, we show that a *Malassezia *secretory protease causes increased inflammation in barrier-compromised skin and has important roles in enabling a planktonic cellular state that can potentially aid in colonization. *Malassezia *secretory proteases alter their external environment through proteolytic cleavage of extracellular host and microbial proteins, and control *Malassezia *cell adhesion. Taken together, our study shows these ubiquitous fungal proteases can be beneficial in healthy individuals but become virulence factors in individuals with a compromised skin barrier.

Skin is our body's first line of defense against the external environment and is also one of the most versatile human organs. Providing essential immunologic and metabolic functions, the skin also serves as a dynamic microbial habitat, allowing the continual exchange of secreted communication molecules between the microflora and host (1). *Malassezia* are a dominant and integral part of the skin microflora and have evolved intimately with the skin to maintain symbiotic host-microbe interactions (2, 3). This unique group of lipophilic commensals is associated with many cutaneous diseases including pityriasis versicolor, seborrheic dermatitis (SD), and atopic dermatitis (AD) (4, 5) and has more recently been found to contribute to non-cutaneous conditions such as Crohn’s disease (6) and pancreatic ductal adenocarcinoma (7). Among the 21 currently accepted species (8), *Malassezia globosa* and **Malassezia* restricta* are by far the most commonly isolated from human skin, with *M. sympodialis* and *M. arunalokei* also common but less often isolated from healthy skin (9). Additionally, *Malassezia furfur* has been frequently identified through culture-based methods in neonates, children, and healthy adults residing in warm tropical climates (10, 11). *M. furfur* can be isolated from multiple human body sites but at a much lower frequency than *M. globosa* and *M. restricta*. Furthermore, *M. furfur* is frequently described in cutaneous disease and accounts for majority of *Malassezia*-associated bloodstream infections (11–13), making the true niche of *M. furfur* unclear.

*Malassezia* secrete a repertoire of hydrolytic enzymes which serve as important mediators of microbial metabolism (14) and microbial-host and inter-microbial interactions (15). Compared to the gut environment which is rich in carbohydrates, the skin is a harsh environment with low pH and poor nutrient sources, with the exception of lipids and proteins (16). Skin microbial enzymes which mediate lipid and protein hydrolysis are therefore likely to have important roles in responding to and shaping the skin environment (17).

Secretory hydrolases can also be hallmarks of pathogenicity, as demonstrated in dermatophytes (18) and *Candida auris* which share similar classes of well-characterized secreted proteinases with *Candida albicans* (19, 20). *Malassezia* are closely associated with SD, a skin condition characterized by erythema and skin flaking in sebaceous skin sites (21, 22). AD is another dermatological condition characterized by skin inflammation and dry pruritic skin that is associated with immunological sensitivity to *Malassezia* (23, 24). Topical application of azoles, pyrithione zinc, and pyrithione derivatives remains the mainstay of treatment to alleviate disease symptoms in both SD and AD (25–29), demonstrating that reduction of *Malassezia* can lead to improvement of dermatological conditions. However, as *Malassezia* are ubiquitously found on human skin, it is unclear what specific molecular components are involved in disease pathogenesis. Despite the relevance of fungal proteases in dermatological conditions, secreted proteases are more extensively studied in bacteria and other lower abundance fungal pathogens (17, 30). In our previous work, we identified the predominant secreted aspartyl protease (SAP) in *M. globosa* which we termed Mgsap1. This enzyme attenuates the formation of biofilms in *Staphylococcus aureus* (31). The homolog in *M. furfur*, Mfsap1, was characterized by its robust catalytic efficiency and wide-ranging substrate preferences including human extracellular matrix (ECM) proteins (32).

In this study, we examined the multifunctional roles mediated by *Malassezia *SAP in modulating fungal-host interactions and homeostatic processes. Using targeted RNA-sequencing of *M. globosa *secretory hydrolases, we observed upregulation of several fungal enzymes regardless of lesional or non-lesional site status in SD and AD patients compared to healthy subjects. Among these enzymes, *MGSAP1 *expression was significantly elevated in both dermatological diseases. To determine the consequence of increased *MGSAP1 *expression and its role in disease pathogenesis, we generated a knockout of the *MGSAP1 *homolog, *MFSAP1*, in the genetically tractable *M. furfur.* We determined that the secretion of Mfsap1 promotes cell dissemination likely through degradation of *Malassezia *cell surface proteins or extracellular polymeric substances (EPS) leading to altered cell surface properties. The loss of *MFSAP1 *abolished free-floating planktonic phase cells and induced a hyper-adherent phenotype on inert matrices and in a reconstructed human epidermis (RHE) model. We further demonstrated that Mfsap1 promotes exacerbation of inflammatory responses in barrier-compromised murine skin through direct interaction of Mfsap1 with the host or potentially via Mfsap1-dependent alteration of fungal cell surface hydrophobicity. Collectively, we show that this SAP in *Malassezia *plays a pivotal role in fungal skin colonization and tissue inflammation when the epidermal barrier is compromised.

## Results

### *M. globosa* Secretory Hydrolase Expression Profiling Reveals Upregulation of MGSAP1 in Atopic and SD Skin.

**Fig. 1.**
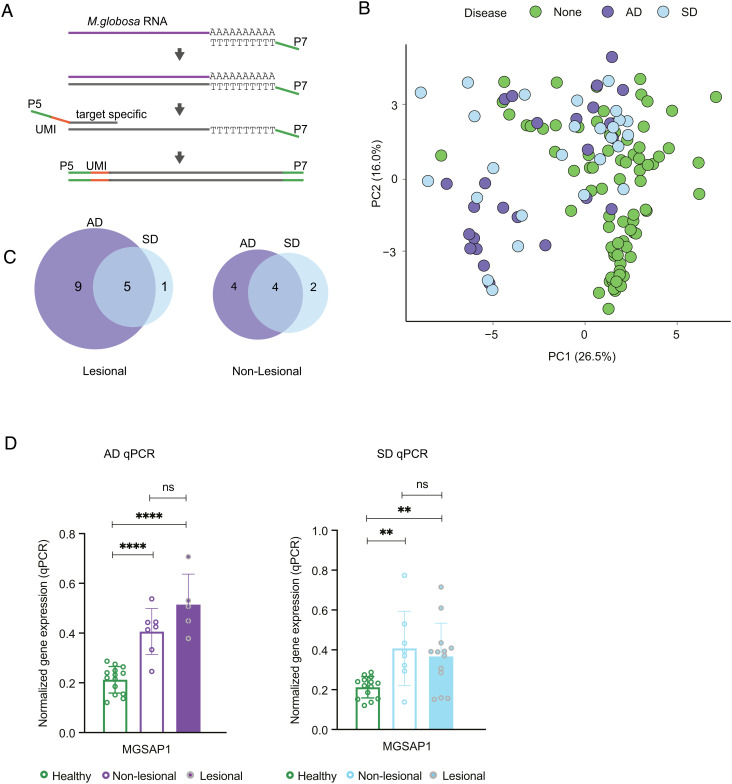
*M. globosa* secretory hydrolase expression reveals a global change in skin environment in dermatological diseases. (*A*) Schematic of the targeted RNA-seq workflow starting from isolated RNA. (*B*) Principal component analysis plot of all *M. globosa* secretory enzyme genes of healthy (green), atopic dermatitis (AD; purple), and seborrheic dermatitis (SD; blue) subjects. For healthy subjects, both the ear and scalp samples were included. For AD and SD patients, both non-lesional (NL) and lesional (L) samples were included. n = 45 for healthy subjects; n = 25 for AD (n = 8 for NL, 17 for L); n = 26 for SD (n = 10 for NL, 16 for L). (*C*) Venn diagram of the differentially expressed genes in AD and SD patients when compared to the healthy subjects. (*D*) Expression of *MGSAP1* (MGL_1932) in healthy versus AD (left) and SD (right), as assessed by RT-qPCR. The same healthy subject data was represented in both panels. Statistics were calculated using one-way ANOVA as appropriate. **P < 0.01, ****P < 0.0001.

We utilized a targeted RNA-sequencing approach that focused on amplification of a panel of *M. globosa* secretory hydrolytic enzymes: 17 extracellular proteases, 13 secretory lipases, six phospholipase C, and four sphingomyelinases, together with two housekeeping genes (Fig. 1*A* and *SI Appendix*, Table S1). After first-strand synthesis with oligo-dT containing the P7 Illumina adaptor, we used a panel of 42 primers designed to have minimal cross-interactions. Each primer contains a unique molecular identifier (UMI) of eight random nucleotides which label individual mRNA transcripts (Fig. 1*A*), reducing amplification bias (33). The library of transcripts was then amplified for Illumina sequencing and the sequencing reads mapped to the library of amplicons using a custom pipeline (*SI Appendix*, Fig. S1). The baseline of *M. globosa *hydrolytic enzyme gene expression was established in 45 healthy volunteers (HV) with no obvious dermatological conditions by non-invasive tape strip sampling of two sebaceous sites—retroauricular crease (behind the ear) and scalp (*SI Appendix*, Table S2) and compared to lesional and non-lesional samples from 19 moderate-to-severe (SCORAD, *SI Appendix*, Table S3) AD patients during acute disease flare and 17 SD patients who were clinically assessed for erythema and adherent scalp flaking severity (*SI Appendix*, Table S4). While it was difficult to match the exact sites sampled on healthy subjects, the sampling sites were focused on sebaceous areas of neck, face, and scalp. Principal component analysis revealed separation of the AD and SD subjects regardless of site lesional status compared to HV (Fig. 1*B*) with no significant difference between the AD and SD groups (*SI Appendix*, Fig. S2). False discovery rate (FDR) analysis of 1% and fold change (FC) of 1.5 or greater identified eight significant differentially expressed genes at non-lesional sites between AD and HV and six between SD and HV. For lesional sites, 14 differentially expressed genes were identified when comparing AD and HV, and six differentially expressed genes when comparing SD and HV (Fig. 1*C* and *SI Appendix*, Fig. S2). For both the lesional and non-lesional sites of AD and SD, there were multiple overlaps in the differentially expressed genes, suggesting that *M. globosa* secretory hydrolase expression was modified in a similar way despite lesional status in both skin conditions. From this differential expression analysis, we observed that *MGSAP1* (MGL_1932), a secretory protease, was significantly upregulated in both the AD non-lesional and lesional sites (FC = 2.3, *P* = 4 × 10^–6^ for non-lesion, FC = 1.8, *P* = 10^–5^ for lesional). We further verified the expression of *MGSAP1* by RT-qPCR and observed significant upregulation of the aspartyl protease *MGSAP1* in both AD and SD lesional and non-lesional sites versus HV. No significant differences were observed between expression of *MGSAP1* between the lesional and non-lesional sites of subjects with either skin disease (Fig. 1*D*).

### Establishing *M. furfur* as Model Organism Using Site-Directed Mutagenesis.

**Fig. 2.**
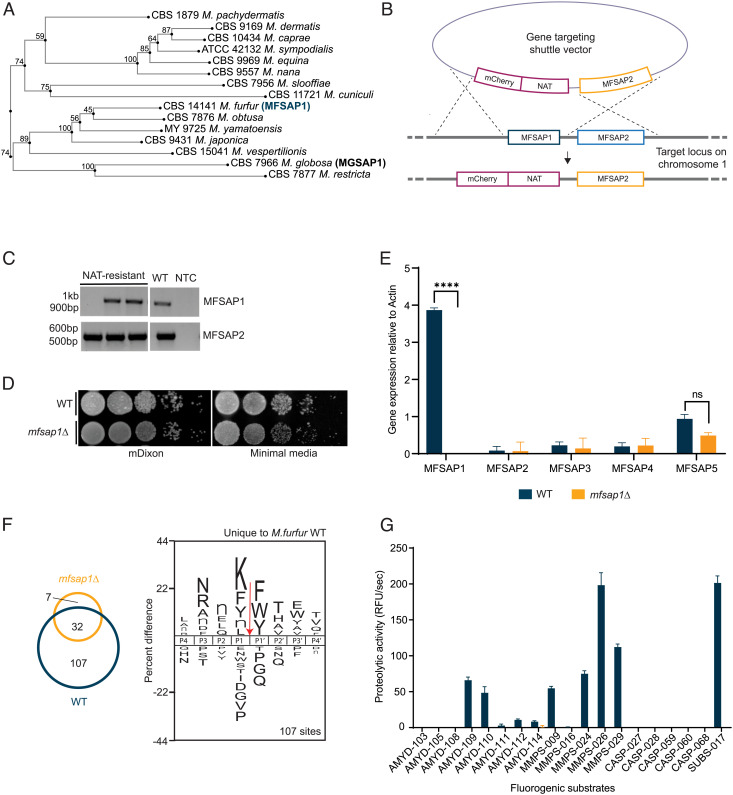
MFSAP1-targeted mutagenesis and functional validation of MFSAP1-deficient mutant. (*A*) Unrooted phylogenetic tree of Mgsap1 homologs in the 15 available *Malassezia* species genomes. Bootstrap values are denoted at each node. (*B*) Homologous recombination-based targeted gene replacement of MFSAP1 gene with mCherry and nourseothricin (*NAT*) markers. MFSAP2 gene was restored in the 3′ homologous arm. (*C*) PCR verification with MFSAP1-specific primers (964 bp amplicon) and MFSAP2-specific primers (528 bp amplicon) against three randomly selected NAT-resistant transformants and wild type (WT) parental strain. NTC, no template control. (*D*) Serial dilution of cell suspension comparing WT and *mfsap1*Δ on mDixon and minimal media. (*E*) Relative gene expression of MFSAP1-5 in WT and *mfsap1*Δ. (*F*) Comparison of cleavage sites from proteases in WT- and *mfsap1*Δ-conditioned media using a library of 228 tetradecapeptides. IceLogo analysis utilized the “positive data set” consisting of the P4 to P4′ amino acids that surround the cleavage sites (red arrow) of the 14-mer peptides and a “negative data set” consisting of the P4 to P4′ amino acids for all 2,964 possible cleavage sites within the peptide library. The amino acid described with lowercase “n” corresponds to Nle, a sulfur-free isostere of methionine. Z-scores were utilized to generate iceLogo illustrations of the relative frequencies of amino acid residues at each of the P4 to P4′ positions of the cleaved peptides where heights of the amino acids represent “percent difference”, defined as the difference in frequency for an amino acid appearing in the positive data set relative to the negative data set. Positive differences are shown above the midline, and negative differences are represented below the midline. (*G*) Comparison of proteolytic activity of WT and *mfsap1*Δ with 19 synthetic fluorogenic substrates. Statistics were calculated using unpaired Student’s *t* test. ****P < 0.0001.

While investigation of *MGSAP1* function and its role in skin disease would be best accomplished by gene deletion in *M. globosa*, this *Malassezia* species is not currently genetically tractable, so a deletion mutant was created in the closely related species *M. furfur* (*mfsap1*Δ). *Malassezia furfur *and *MFSAP1* were chosen as Mfsap1 is more closely related to Mgsap1 than homologs in the other tractable *Malassezia* species, *M. sympodialis* (Fig. 2*A*). Mfsap1 shares 68% similarity to Mgsap1 at the protein level and is the most predominantly expressed SAP of the five identified *M. furfur* SAP *in vitro*, herein named as *MFSAP2* (FUN_000222), *MFSAP3* (FUN_001776), *MFSAP4* (FUN_003258), and *MFSAP5* (FUN_003259) (*SI Appendix*, Fig. S3 and Table S5) (32). Also, the proteolytic activity of secreted Mfsap1 shared a comparable endoproteolytic profile with Mgsap1 (32).

A gene deletion mutant of the *M. furfur MGSAP1* homolog, FUN_000223, or *Malassezia furfur* SAP 1, *MFSAP1* (32), was generated in *M. furfur* CBS14141 wild type (WT). Targeted gene deletion was performed with *Agrobacterium tumefaciens*-mediated transformation by replacing the *MFSAP1* gene with codon-optimized mCherry as a fluorescent tag and nourseothricin (*NAT)* as the selection marker under the regulatory control of *M. sympodialis* actin promoter and terminator. As the *MFSAP2* gene is immediately adjacent to the 3′ end of *MFSAP1*, the entire *MFSAP2* coding frame was contained in the 3′ homologous recombination arm (Fig. 2*B*). PCR detection and Sanger sequencing showed accurate homology-directed repair and preservation of the flanking gene sequences, including the complete predicted *MFSAP2* gene upstream sequence and open reading frame (Fig. 2*C*). Spot assays confirmed that both WT and *mfsap1*Δ showed comparable growth and viability with no obvious impact on cell fitness in rich and minimal media (Fig. 2*D*). Using RT-qPCR of RNA isolated from *in vitro M. furfur* cultures, we determined that *MFSAP1* was the most highly expressed of the identified SAPs in WT, with low to negligible expression of the remaining SAPs (Fig. 2*E*). In *mfsap1*Δ, there was a complete absence of *MFSAP1* expression (Fig. 2*E*) with no significant change in *MFSAP2*, *3*, *4*, and *5* expression, indicating deficiency of *MFSAP1* did not lead to compensatory expression of other SAPs. To compare the extracellular protease activity in *M. furfur* WT and *mfsap1*Δ, we generated cell-free conditioned media from 48-h cultures of each strain and incubated with a library of 228 synthetic peptide substrates containing diverse sequences of 14 residues. This peptide library was rationally designed to contain substrate sequences for all classes of proteases (34, 35) and its utility for uncovering the substrate specificity profile of the previously characterized Mgsap1 (31, 32), and other fungal aspartyl proteases have been previously demonstrated (36, 37). When proteases in the media cleaved these peptides, the exact cleavage site was determined using mass spectrometry and the amino acids at each side of the cleaved bond were revealed. Proteases in the WT media cleaved at 139 sites while proteases in *mfsap1*Δ media cleaved at only 39 sites. In total, 107 sites were cleaved by proteases in WT media and not by proteases in *mfsap1*Δ media (Fig. 2*F*). Therefore, these 107 sites are likely to be cleaved by Mfsap1. We generated a substrate specificity profile of the 107 cleavages sites corresponding to the P4 to P4′ residues which revealed that MfSAP1 has a strong preference for cleavage on the C-terminal side of Lys, Phe, Tyr, norleucine (Nle), and Leu and on the N-terminal side of Phe, Trp, and Tyr (Fig. 2*F*). At other sites, Nle is preferred at P2 while Asn, Arg, and Ala are preferred at P3. Overall, this substrate specificity profile is highly comparable to the profile that we have previously generated for MgSAP1 using the same set of 228 peptide substrates (32). To validate the loss of protease activity in the *mfsap1*Δ-conditioned media, we also incubated the WT and *mfsap1*Δ media with a panel of 19 fluorogenic substrates that we had previously used to screen for protease activity (*SI Appendix*, Fig. S4). Proteases in the WT media efficiently cleaved 10 substrates; however, this activity was reduced to baseline in the *mfsap1*Δ-conditioned media (Fig. 2*G*). These data demonstrate that MfSAP1 is the major protease secreted by *M. furfur*.

### Loss of MFSAP1 Abolishes the Planktonic Phase and Promotes Accumulation of Sessile Cells through Alteration of Cell Surface Properties or Protein Abundances.

**Fig. 3.**
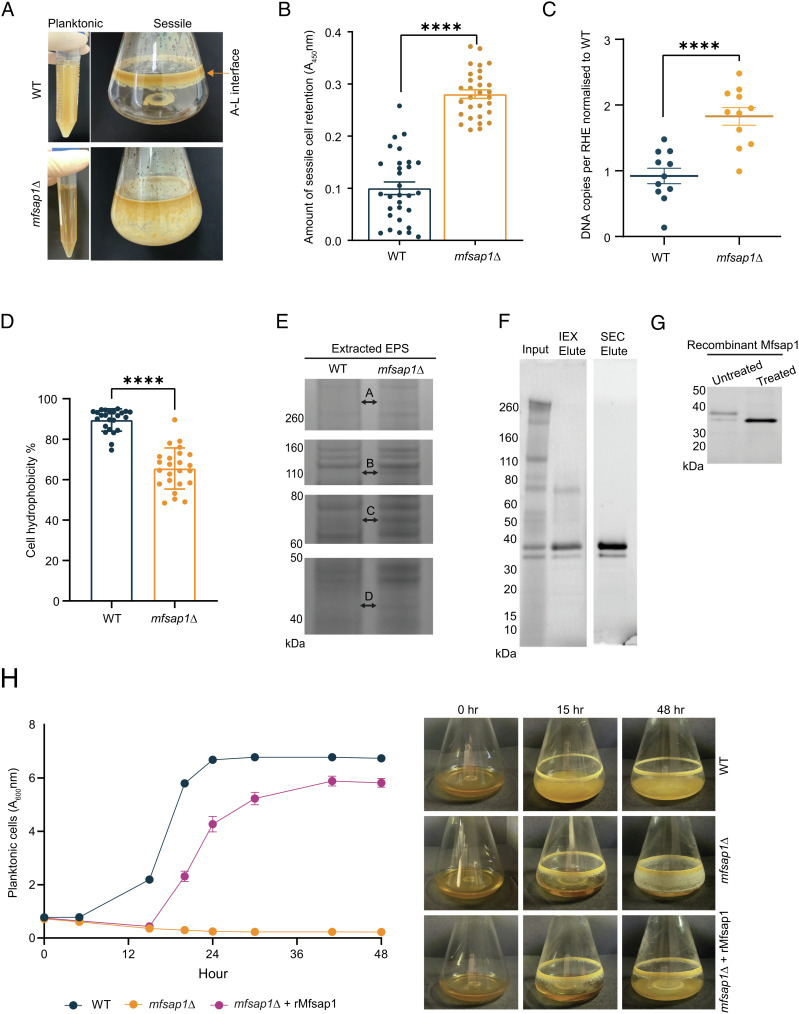
Mfsap1 positively regulates cell dispersal from hyperadherent cells and modifies cell surface properties. (*A*) WT parental strain generates planktonic (free-floating) cells and sessile (highly adherent) cells at the air-liquid interface. The deficiency of Mfsap1 enables the accumulation of sessile cells with an absence of planktonic cells in *mfsap1*Δ. (*B*) Relative quantification of retained sessile cells after repeated washes with an XTT metabolic activity assay. (*C*) Absolute quantification of *M. furfur*-specific internal transcribed spacer (ITS) rDNA in *mfsap1*Δ and WT-infected RHE. (*D*) Microbial adhesion of WT and *mfsap1*Δ cells to aliphatic hydrocarbons. (*E*) SDS-PAGE analysis of the WT and *mfsap1*Δ extracellular polymeric substances. Four representative bands of the SDS-soluble proteins (labeled A–D) were detected mainly in *mfsap1*Δ extract and were excised and identified via MS/MS. (*F*) Recombinant Mfsap1 was sequentially purified with cation-exchange and size exclusion chromatography. (*G*) Purified recombinant Mfsap1 contains glycosylated and non-processed forms of the protein. (*H*) Protein-based complementation assay effectively rescues planktonic state in *mfsap1*Δ. Statistics were calculated using Student’s *t* test. *P < 0.05, ****P < 0.0001.

Several *Malassezia* strains are recognized to be active producers of extracellular polymeric substances (EPS) that contributes to fungal virulence and pathogenesis (38–41). Most *Malassezia* species, including *M. furfur* CBS14141 WT, can simultaneously produce planktonic and sessile cells at an air-liquid (A-L) interface when broth cultures are maintained with agitation (Fig. 3*A*). *mfsap1*Δ displayed a dramatically altered sessile phenotype with an accumulation of sessile cells in a dense A-L interface and almost complete absence of planktonic cells (Fig. 3*A*). We assessed the adhesion of sessile cells through measuring the number of retained cells after repeated washes with an XTT metabolic activity assay. *mfsap1*Δ demonstrated stronger cell adherence to polystyrene plastic through significantly higher cell retention as compared to the parental strain (Fig. 3*B*).

To assess whether Mfsap1 plays a role in facilitating adhesion to human skin, we utilized a 3D RHE model with *Malassezia* colonization. WT or *mfsap1*Δ cells were topically applied to barrier intact RHE. Absolute quantification of *M. furfur*-specific internal transcribed spacer (ITS) rDNA showed higher ITS copy number in *mfsap1*Δ-infected RHE than in the WT-infected counterparts (Fig. 3*C*). Both WT and *mfsap1*Δ *Malassezia* cells were present only on the apical surface and did not invade into the deeper epidermal layers (*SI Appendix*, Fig. S5*A*). There was a trend but not a statistically significant increase of IL-1RA, IL-1β, IL-6, IL-8, and TNFα cytokines in response to *M. furfur* infections while, IL-1α and IL-10 were not detected reproducibly after exposure to either strain (*SI Appendix*, Fig. S5*B*).

We next assessed if cell surface hydrophobicity was altered in relation to cell adhesion by measuring the microbial adhesion to hydrocarbons (MATH) (42). WT cells were significantly more hydrophobic (89.5% ± 5.54%) than *mfsap1*Δ cells (65.6% ± 10.2%; *P* < 0.0001) (Fig. 3*D*). To further investigate the potential substrates of Mfsap1 that result in the difference in cell adhesion and surface hydrophobicity, *Malassezia* EPS were extracted following a previous method established in *C. albicans* (43) from WT and *mfsap1*Δ sessile cells on the A-L interface. EPS protein extracts were first separated on SDS-PAGE, and four distinct protein fractions (bands labeled A–D) were observed in *mfsap1*Δ (Fig. 3*E*). These protein bands were excised and in-gel tryptic digested before LC-MS/MS analysis. The identified proteins were further analyzed and functionally shortlisted for secreted proteins with >1.5-FC and subsequently screened for predicted preferred cleavage sites based on tetradecapeptides protease substrate profiling data. Although the overall proteome differences in these fractions between *mfsap1*Δ and the parental strain were modest, we saw the accumulation of two putative extracellular proteins including cadherin-like protein (FUN_000076) and Hsp70 (FUN_002744) in *mfsap1*Δ (Fig. 3*E*).

To further confirm the role of Mfsap1 in facilitating cell detachment, we expressed recombinant Mfsap1 in *Pichia pastoris* and purified using cation-exchange followed by size-exclusion chromatography. This resulted in isolation of two recombinant protein products between 35 and 40 kDa (Fig. 3*F*), similar to the previously reported pepstatin A-agarose pull-down of native MfSAP1 from WT culture supernatant (32). Enzymatic deglycosylation of the purified Mfsap1 protein generated a single protein band with a shift in electrophoretic mobility to approximately 35 kDa (Fig. 3*G*). In order to determine if the fully sessile *mfsap1*Δ phenotype can be reversed to planktonic growth, we performed complementation experiments over 48h with either WT-conditioned medium (CM) or purified recombinant Mfsap1 protein (Fig. 3*H*). *mfsap1*Δ liquid cultures were supplemented with 15% WT-CM, the equivalent amount of recombinant Mfsap1 protein (rMfsap1, 5.22 μg), or 15% of *mfsap1*Δ-CM as control (Fig. 3*H* and *SI Appendix*, Fig. S6). Over 48h, there was a time-dependent increase of planktonic cells in rMfsap1 (25-fold) and WT-CM (16.5-fold) treated flasks (Fig. 3*H* and *SI Appendix*, Fig. S6). Only complementation with purified rMfsap1 or *M. furfur* WT-CM was able to rescue the release of planktonic cells from the enhanced sessile A-L interface phase in *mfsap1*Δ.

### Mfsap1 Promotes an Inflammatory Response in a Murine Skin Model of AD.

**Fig. 4.**
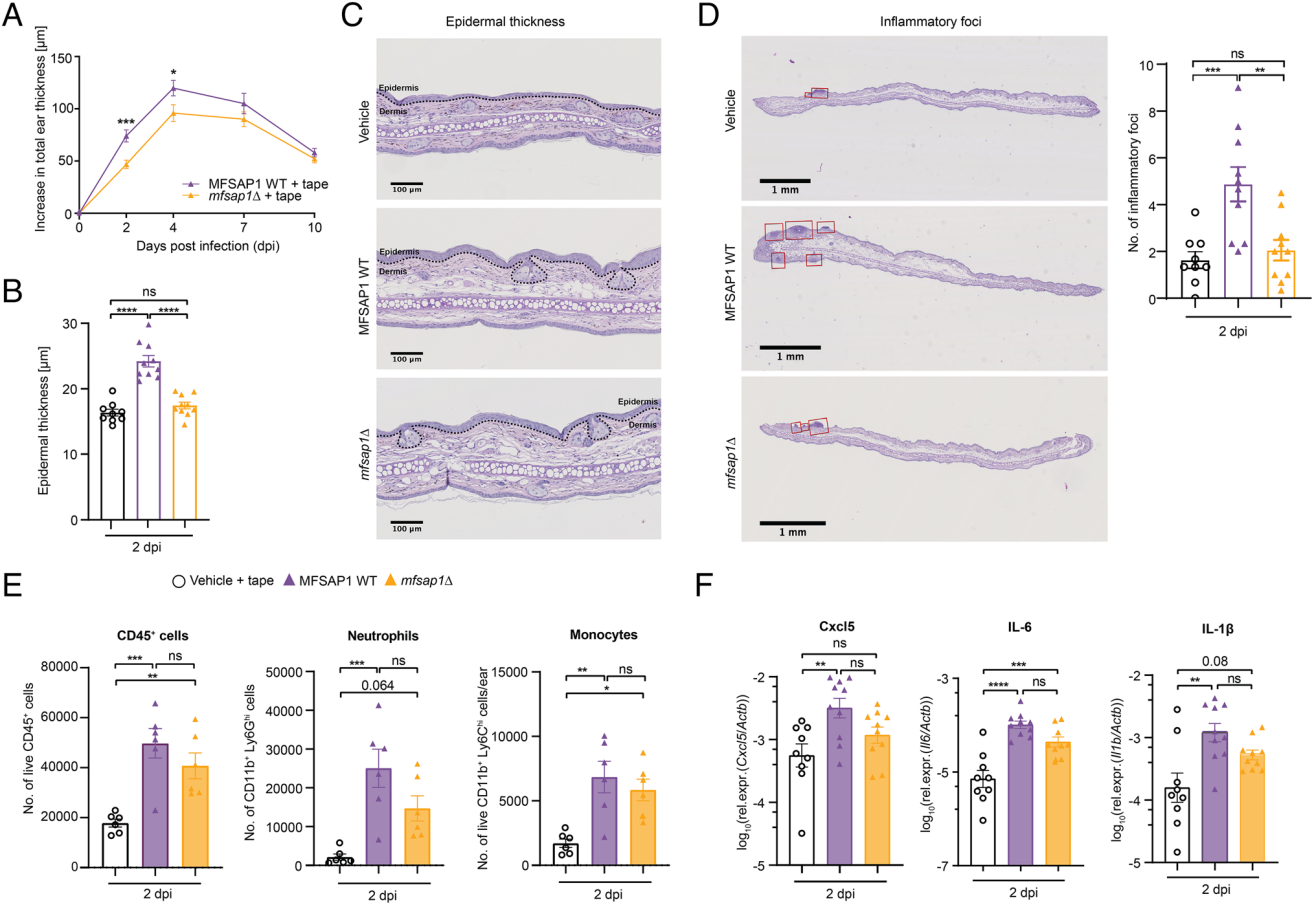
Mfsap1 promotes an inflammatory response in barrier-compromised skin. C57BL/6 wildtype mice were tape stripped and epicutaneously infected with approx. 5 × 10^6^ MFSAP1 WT (Mf::mc-27) or *mfsap1*Δ cells dissolved in olive oil and ear skin was analyzed at 2 and 10 d post-infection (dpi). (*A*) Ear swelling measured with a caliper over the course of 10 d. (*B*) Epidermal thickness quantified in periodic acid-Schiff (PAS)-stained histological sections at 2 dpi. (*C*) Representative PAS-stained histological section at 2 dpi. (*D*) Quantification of inflammatory foci (myeloid infiltrates) per histological ear section taken from three independent experiments with an average of three slides per mouse (total = 9–10 mice per group). (*E*) Quantification of myeloid ear infiltrates by flow cytometry at 2 dpi. Number of live CD45^+^ cells, CD11b^+^ Ly6C^hi^ Ly6G^-^ monocytes and CD11b^+^ Ly6G^+ ^neutrophils per ear. (*F*) Cxcl5, IL-6, and IL-1β gene expression detected by RT-qPCR with whole mouse skin extracts; one data point of IL-6 from *mfsap1*Δ-infected mice was excluded due to non-detectable C_T_value. Data were pooled from three independent experiments with 3–4 mice per group each for Fig. 4 *A*–*D* and *F*. Flow cytometry in Fig. 4*E* was performed in two independent experiments with three mice per group each. Representative histology sections were taken for each experimental group in Fig. 4 *C* and *D*. Statistics were calculated using Student’s *t* test (Fig. 4*A*) or one-way ANOVA with Šídák's multiple comparisons test (Fig. 4 *B*–*F*). *P < 0.05, **P < 0.01, ***P < 0.001, ****P < 0.0001.

To study the effect of Mfsap1 on the interaction with the host in vivo, we utilized the previously established *Malassezia* murine infection model (44). The skin barrier was disrupted by mild tape stripping prior to colonization to mimic conditions characteristic of AD, under which *Malassezia* induce an exacerbated inflammatory response in the skin (44). The Mf::mc-27 strain was used as a control for *mfsap1*Δ, herein referred to as MFSAP1 WT, as it has equivalent cellular fitness and expression of Mfsap1 relative to the parental strain and simultaneously expresses mCherry analogous to *mfsap1*Δ (45). Fungal loads on murine skin were high on day 2 post-infection (p.i.) and still evident on day 10 p.i., without differences between Mfsap1-deficient and Mfsap1-sufficient *M. furfur* strains (*SI Appendix*, Fig. S7*A*). Lack of Mfsap1 resulted, however, in a diminished inflammatory response, assessed as swelling of the infected ear. The effect was most pronounced on day 2 and day 4 p.i. (Fig. 4*A*). Consistently, epidermal thickness assessed via histological sections revealed increased epidermal hyperplasia in MFSAP1 WT-infected mice as compared to both control and *mfsap1*Δ-infected mice (Fig. 4 *B* and *C*). Epidermal thickness in *mfsap1*Δ-infected mice was not significantly increased when compared to uninfected controls (Fig. 4 *B* and *C*). This potential Mfsap1-dependent response was not only reflected in overall increased ear swelling due to edema, but also in a more pronounced appearance of myeloid infiltrates (inflammatory foci), which were induced in larger numbers in mice infected with MFSAP1 WT than with *mfsap1*Δ strain (Fig. 4*D* and *SI Appendix*, Fig. S7*B*). The reduction in inflammatory infiltrates, in particular infiltrating neutrophils, was also observed in infected ear skin although differences between Mfsap1-deficient and Mfsap1-sufficient strains did not reach statistical significance as quantified by flow cytometry in skin single-cell suspensions (Fig. 4*E* and *SI Appendix*, Fig. S7*C*). Similarly, despite nonsignificant differences relative to MFSAP1 WT, the attenuated inflammatory response to *mfsap1*Δ was further reflected in a slightly reduced induction of neutrophil attracting chemokine Cxcl5 and inflammatory cytokines IL-1β and IL-6 assessed by RT-qPCR of whole skin extracts (Fig. 4*F*). This further supports the role of Mfsap1 in promoting inflammation in compromised host skin. In contrast, activation of the IL-17 pathway and synergistic induction of β-defensin 3, which are critical for fungal control but uncoupled from neutrophil recruitment to the colonized skin (44), were not affected by the absence of Mfsap1 (*SI Appendix*, Fig. S7*D*).

## Discussion

Our skin microbiome is responsive to changes in micro- and macro-environments which subsequently influence the diversity and abundance of the local microbial communities. It is necessary for members of the microbial community and the host to communicate and interact. The role of cutaneous commensal secretory hydrolases remains underexplored, although the direct microbe-epithelial cell contact, distribution of microbial by-products, and secreted factors likely have considerable effects on skin. In this study, we examined the role of an aspartyl protease released by *Malassezia* which has functional relevance on human skin. Overall, we observed that the expression of this secreted hydrolase can change markedly in AD and SD patients compared to healthy individuals. In addition, this enzyme controls fungal cell adhesion and dispersal and is a notable driver of inflammation in barrier compromised skin, suggesting that Mfsap1 is instrumental in influencing skin health.

We first focused on *M. globosa* as it is prevalent and abundant on mammalian skin, and the role of secreted lipases in SD is well defined (29, 46–48), but the role of other secreted factors in host and inter-microbial interactions is not well understood. *Malassezia* have been associated with several skin conditions, in particular SD, even though *Malassezia *abundance in cross-sectional studies is poorly correlated with SD disease severity (12). Furthermore, the reduction of *Malassezia* populations correlates with the improvement of flaking severity in individuals affected by dandruff, even predicting anti-flaking efficacy (49, 50). Previous studies have shown that there is an underlying skin barrier permeability dysfunction in SD and AD patients, and the barrier impairment is not confined to lesional areas (51). Our study further supports this key finding with the detection of increased expression of the previously characterized Mgsap1 protease (31) in both the lesional and non-lesional sites in AD and SD patients as compared to healthy individuals. This suggests that this secretory hydrolase is not directly involved in driving pathogenesis, but expression is affected by the abnormal skin environment or stratum corneum. The altered patient epidermal barrier properties (52) could make them more susceptible to penetration and sensitization to these enzymes. Additionally, the potential involvement of other microbial interactions may also work in an additive manner with Mgsap1 in exacerbating the clinical phenotypes (53). We have recently shown that Mfsap1, the homolog of Mgsap1 found in *M. furfur*, is able to degrade ECM proteins associated with the dermis and epidermis (32). With increased barrier permeability, we postulated that this protease could degrade host ECM proteins and further break down the skin barrier in patients with altered stratum corneum structures. This is consistent with recent studies demonstrating the role of other microbial extracellular proteases in inducing skin inflammation during AD (17).

Detailed examination of the role of MGSAP1 in pathogenesis would be most directly facilitated by gene deletion. However, *M. globosa*, likely due to its fastidious growth conditions, has not yet been amenable to genetic manipulation. *M. furfur*, a closely related species, has previously proven to be a competent model organism with high phenotypic robustness and moderate transformation efficiency (45, 54). Therefore, we identified and replaced the MGSAP1 gene homolog in *M. furfur* strain CBS14141, MFSAP1, with mCherry and *NAT* selection markers. *mfsap1*Δ displayed differential growth and significant lack of proteolytic activity with no apparent genetic compensation or significant differential gene expression of the other MFSAPs. *In vitro*, the lack of any growth defect and absence of a compensatory response in *mfsap1*Δ may indicate that this protease is not a key enzyme in nutrient acquisition, a function commonly thought to be a major role of secretory fungal proteases (55, 56). Instead, our work demonstrates that this protease plays essential roles in facilitating cell detachment from the sessile phase to enable cell dissemination. Treatment of *mfsap1*Δ cultures with purified recombinant Mfsap1 protein or WT-CM containing comparable quantity of native Mfsap1 resulted in a time-dependent recovery of planktonic growth, while there was no restoration of planktonic growth in *mfsap1*Δ when treated with its own CM. These data suggest secretion of Mfsap1 positively modulates cell dispersal from the sessile phase through targeting extracellular proteins or polymeric substances on the cell wall. While there are modest differences between *mfsap1*Δ and the parental strain EPS proteome profile, we detected the accumulation of putative cadherin-like protein and heat shock protein 70 (Hsp70) which may serve as potential substrates of Mfsap1. Cadherin-like proteins and Hsp70 were previously identified as important components of microbial biofilm matrices and contributors of cell adherence to host cells (57, 58).

Variations of extracellular proteins can lead to significant exterior modifications which influences cell surface hydrophobicity. The production of Mfsap1 potentially enabled WT cells to become more hydrophobic. In comparison, *mfsap1*Δ is significantly more hydrophilic, potentially contributing to a directional shift from free-floating to adherent sessile cells. Additionally, *mfsap1*Δ showed high cell adsorption efficiency on abiotic surfaces including plastic as well as greater colonization on epithelial surfaces of RHE. Given that both the WT and mfsap1Δ have similar growth rates (Fig. 2*D*), the difference in abundance on the RHE is likely due to differences in adhesion. Cell surface hydrophobicity has been shown to influence microbial biophysical properties and virulence, with *Malassezia* hydrophobicity linked to stimulation of proinflammatory mediator expression in keratinocytes and human peripheral blood mononuclear cells (59, 60). In our study, *Malassezia*-infected RHE showed a negligible response of IL-1RA, IL-1β, IL-6, IL-8, and TNFα cytokine upregulation. As RHE are exclusively made up of keratinocytes and hence unable to recapitulate the cellular complexity of the skin, we utilized an established murine infection model that showed the promotion of *Malassezia*-induced inflammation in (tape-stripped) barrier-compromised skin (44). We identified Mfsap1 was an important driver of this effect, which was most evident during the early time points of analysis (day 2 p.i.). The effect manifested in increased ear swelling, hyperplasia, and the more pronounced myeloid infiltrates induced by WT control. In contrast, *mfsap1*Δ-infected mice resembled uninfected controls, presenting an attenuated inflammatory response further corroborated by the diminished Cxcl5-mediated neutrophil recruitment and IL-1β and IL-6 inflammatory markers.

Collectively, we have demonstrated that the major *Malassezia* SAP plays significant roles in influencing skin colonization and inflammation especially in compromised skin. Unlike its closest phylogenetically related plant pathogen ancestor, *Malassezia* share higher similarities to *Candida* and *Cryptococcus* in the release of hydrolases required for host tissue invasion and epithelial colonization (19, 61). While *Candida* and *Cryptococcus* predominantly affect immunocompromised individuals, the secretion of proteases allows *Malassezia* to effectively inhabit and invade the human host simultaneously (47). Additionally, this work, together with previous studies on other fungal aspartyl proteases, demonstrates that this class of enzyme is crucial mediator of host-fungal interactions (17, 56). Mfsap1 is capable of directly altering the external environment through proteolytic cleavage of extracellular protein belonging to both the host and other microbes (31, 32), has a critical role in controlling *Malassezia* cell adhesion by modifying cell surface properties, and plays a role in induction of inflammation. This study shows that enzymes secreted by “commensal” microbes could be beneficial in healthy individuals (as demonstrated by the role of Mgsap1 in bacterial biofilm degradation) but become virulence factors in the context of individuals with compromised skin barrier. Taken together, our study highlights the importance of studying skin microbial community secretory enzymes and to examine their functions in the context of both healthy and weakened cutaneous barrier.

## Materials and Methods

### Skin Microbiome Sampling of Healthy Subjects and Skin Disease Patients.

Skin tape strip sampling for HVs was approved by the Institutional Review Board of the National University of Singapore under B-15-237 and all subjects provided written consent before participation. Inclusion and exclusion criteria, HV sampling, and sample processing follow the same procedures as we have reported previously (*SI Appendix*, Table S2) (31). Sampling of skin disease patients was approved by the National Healthcare Group Domain Specific Review Board under 2017/00033. All healthy subjects were sampled at the left and right retroauricular crease and frontal scalp. The left and right tape strip samples were stored in bead-beating tubes with TRIzol at –80°C separately and combined at the RNA extraction step. For skin disease patients, subjects were recruited during their routine clinic appointment by the attending physician and written consent was obtained. Only adult patients >21 y old were included in both the AD and SD study. Patients on oral retinoid, topical corticosteroid, and topical anti-fungal were excluded from the study. AD patient disease severity was assessed using SCORing AD (SCORAD), and SD patient severity was assessed using Adherent Scalp Flaking Severity (62) and Clinical Scalp Erythema Score. Metadata associated with each patient including sampling sites is included in *SI Appendix*, Tables S3 and S4. Patients were sampled at two control (non-lesional) sites and two diseased (lesional) sites with D-SQUAME® tape strips (Clinical and Derm, USA) using the same method as for healthy subjects and samples were frozen at –80°C. For RNA extraction, 1 mL TRIzol reagent was added to the tape stripes and frozen at –80°C until further processing. The two control sites were combined at the RNA extraction step, as were the two sampled diseased sites. RNA extraction from the tape strips was performed as previously described (31).

### Targeted RNA-seq Library Preparation.

5 μL of sample RNA was used to construct the sequencing library using the QuantSeq-Flex Targeted RNA-Seq Library Prep Kit V2 following the manufacturer’s protocol (Lexogen, Austria) with several modifications. For first-strand synthesis, incubation was performed for 60 min at 42°C. For second strand synthesis, 2 μL of the 0.5 μM pooled primer mix containing 47 primers was added to 7 μL of the mastermix (TS) and 1 μL of Enzyme Mix (E). This 10 μL reaction was added to the first-strand synthesis reaction, and incubation was performed for 2 min at 98°C, 15 mins at 54°C, 20 min at 72°C, and temperature held at 10°C. The cycle number for amplification was determined using the PCR Add-on Kit for Illumina (Lexogen). For RNA isolated from tape strips, the samples were amplified for 28 cycles. Concentration of the sequencing library was determined using the Qubit dsDNA HS assay kit (Thermo Fisher Scientific). The library size was assessed using the Bioanalyzer HS DNA kit with Bioanalyzer (Agilent, Singapore). List of primers for targeted RNA-seq (second strand synthesis) is in *SI Appendix*, Table S1. All primers for the second-strand synthesis were designed with the following structure—5′ P5 adaptor sequence (according to manufacturer’s protocol), eight nucleotides unique molecular index, and a short sequence priming our genes of interest. The sequence of the region corresponding to each gene of interest was designed using PrimerBlast to be selective for *M. globosa* CBS7966 and not target any sequences from human, *Malassezia sympodialis*, *Propionibacterium *(*Cutibacterium*) *acnes*, *Staphylococcus epidermidis*, and *S. aureus*. The 47 sequences were then checked using Multiple Primer Analyzer (ThermoFisher Scientific) to minimize cross-dimers. The average size of the amplicon excluding the sequencing adaptors, 3′ UTR, and poly-A tail is 154 bp (range 77–198 bp). This library of transcripts was then amplified for Illumina sequencing and the sequencing reads mapped to the library of amplicons using a custom pipeline (*SI Appendix*, Fig. S1).

### Analysis and Quantification of Barcoded Sequenced Reads.

All sequencing data were deposited under BioProject Accession ID: PRJNA658716. Sequenced read using Lexogen library preparation method was processed using UMITOOLs (63). The analysis adhered to the workflow illustrated at (https://github.com/CGATOxford/UMI-tools/blob/master/doc/QUICK_START.md). In general, the analysis workflow begins with extracting UMI sequences from raw reads, followed by mapping of the reads onto a custom BWA amplicon reference consisting of the collection of targeted amplicon FASTA sequences. Alignment of these reads was performed using BWA (64). SAMtools (65) was used to index and sort the mapped BAM file from BWA. A final de-duplication step based on the UMI was then performed on these mapped reads to obtain a quantitative estimation of abundance for each of the amplicons. The number of reads associated with each of the amplicon were obtained using samtools idxstats. Samples were ran in parallel using GNU parallel (https://www.gnu.org/software/parallel/). Read depth normalization was performed on the number of amplicons for each target by division of total reads to obtain the fractional contribution of each amplicon scaled to a million. The sample bash script for running a batch of samples is attached in *SI Appendix*, Table S8.

### Data Analysis for Differentially Expressed Genes.

Using the UMI-collapsed counts, all genes were first normalized by the total count of each sample (total of 42 genes). As fragment size of all the targeted genes was similar (min: 77 bp, max: 198 bp, mean: 153 bp), no further normalization for gene length was performed. Statistically analysis was performed using Prism 8.0 using multiple T-test with FDR <1%. We defined differentially expressed genes by using FDR of <1% and FC of 1.5 or greater between healthy subjects and skin disease patients. Principle component analysis (PCA) and heatmaps with hierarchical clustering were generated using R version 4.0.0.

### Strains and Culture Conditions.

*M. furfur* CBS14141 was obtained from the Westerdijk Fungal Biodiversity Institute. Cells were cultured at 32°C, with mDixon medium (36 g/L malt extract, 20 g/L desiccated oxbile, 6 g/L peptone, 2 mL/L glycerol, 2 mL/L oleic acid, 10 mL/L Tween 40, and pH 6). Alternatively, minimal medium was used (3.4 g/L YNB without amino acids or ammonium sulfate, 5 g/L ammonium sulfate, 2 mL/L glycerol, 10 mL/L Tween 40, and pH 6). For standard shake-flask method, 12 mL cultures in 125-mL vented Erlenmeyer flasks (Sigma Aldrich) were maintained at 32°C, 150 rpm. Microtiter plates were sealed with adhesives film (4titude), kept in static incubator at 32°C.

### MfSAP1-Targeted Mutagenesis.

MFSAP1 gene deletion construct was first assembled using previously established protocol (66). Briefly, 1.5 kb upstream and 2.55 kb downstream of MFSAP1 gene (*SI Appendix*, Table S6) were PCR amplified with high fidelity polymerase (Takara). The 2.55 kb downstream flanking arm also contains MFSAP2 gene ORF to avoid untargeted double gene displacement. The third PCR fragment included the nourseothricin (*NAT*) antibiotic resistance and mCherry genes under the regulatory actin promoter sequence from pJG201702 (45). The 4 DNA fragments were combined and assembled in *S. cerevisiae* with lithium acetate (67). Correctly constructed plasmid was verified with PCR screening and Sanger sequencing before plasmid DNA was transferred to *E. coli* to generate higher plasmid yield. Lastly, resultant MFSAP1 knockout vector was electroporated into EHA105 *A. tumefaciens* and verified using PCR and restriction digestion. *Agrobacterium*-mediated transformation of *M. furfur* WT was carried out using previously outlined protocol (45, 66). In brief, *A. tumefaciens* containing MFSAP1 knockout vector was grown in induction medium containing 100 µM acetosyringone (Sigma) and *M. furfur* CBS14141 WT were harvested at log phase. Equal proportions of *Malassezia* and *Agrobacterium* cells were mixed thoroughly and passed through 0.45-µm mixed cellulose membrane (Merck, Millipore) and transferred onto induction media agar supplemented with 200 µM acetosyringone. Cells were incubated at room temperature for 5 d before cells were washed in 20 mL sterile PBS and transferred onto mDixon with 100 µg/mL *NAT*, 200 µg/mL cefotaxime, and 10 µg/mL tetracycline.

### Molecular Analysis.

To extract genomic DNA from *Malassezia*, cells were processed with a combination of two extraction kits. Yeast cells were first suspended in yeast cell lysis solution using MasterPure Yeast DNA kit (Epicentre) and homogenized (BioSpec) for 1 min at maximum speed for three times. Samples were centrifuged at 15,000 g for 3 min followed by RNase A treatment. Subsequently, equal volume of MPC protein precipitate solution was added and cell debris were collected by maximum centrifugation. Supernatant were transferred and added with Buffer AL from DNeasy Blood and Tissue kit (Qiagen). Consecutive purification steps were performed according to the manufacturer’s protocol (Qiagen). Total RNA extractions were performed with TRIzol reagent and Direct-Zol RNA Miniprep kit (Zymo Research) as per manufacturer’s protocol including additional steps of complete DNA removal using Turbo-DNase (Thermo Fisher Scientific) and homogenization at maximum speed for 1 min, repeated thrice. First-strand cDNA pool was synthesized with random hexamer primers and the SuperScript III Reverse Transcriptase kit (Thermo Fisher Scientific). Quantitative PCR was performed with LUNA universal qPCR mastermix (NEB, USA) according to manufacturer’s fast ramp protocol on the Applied Biosystem StepOne Plus Real-Time PCR System (Thermo Fisher Scientific). List of *M. furfur* specific primers were first assessed to have primer efficiency of >90% (*SI Appendix*, Table S5).

### Cloning and Purification of MfSAP1 Recombinant Protein.

MFSAP1 gene was PCR amplified and cloned in *Pichia pastoris* GS115 following the condensed transformation method (68). Culture supernatants were collected after 72 h after methanol induction, and native Mfsap1 was enriched using Amicon 30 kDa ultra-centrifugal filter (Merck Millipore) and buffer exchanged into 20 mM sodium acetate buffer pH 4.2. Protein was subsequently purified through ion-exchange chromatography with HiTrap SP-HP cation exchange column (GE Healthcare) and size-exclusion chromatography with HiPrep 16/60 Sephacryl S-100 HR column (GE Healthcare). SEC fractions ranging from 25 and 45 kDa were collected and enriched using Amicon 10 kDa ultra-centrifugal filter (Merck Millipore) before protein was aliquoted and snap-freeze in liquid nitrogen and stored in −80°C.

### FRET-Based Proteolytic Assay.

Clarified culture supernatants were diluted in 50 mM sodium citrate buffer pH 4.2 to a final concentration of 2% (v/v). A total of 19 generic fluorogenic substrates (CPC Scientific) (*SI Appendix*, Fig. S4) were added to the final concentration of 20 μM. Fluorescence was measured at Excitation/Emission = 330/390 nm on BioTek Synergy H1 microplate reader, and proteolytic activity was calculated as a change in relative fluorescence units per sec using the slope of the linear range for this signal.

### Aspartyl Protease Substrate Profiling.

Conditioned media (0.1 µg/mL) was incubated with an equimolar mixture of 228 14-mer peptides, each at 0.5 μM, in 50 mM sodium citrate pH 4.2. The design of this library has been previously described (34, 35). After incubation for 15 and 60 min at 25°C, 20 μL aliquots were removed and combined with 80 μL of 8 M urea to inactivate the enzymes. An inactivated enzyme control consisted of media in assay buffer mixed with 8 M urea followed by addition of the peptide library. All assays were conducted in quadruplicate reaction tubes. After incubation, samples were acidified by addition of 40 μL of 2% trifluoroacetic acid (TFA), desalted using C18 spin columns, evaporated to dryness in a vacuum centrifuge, and stored at −80°C. Samples were resuspended in 40 μL of 0.1% TFA (solvent A), and 4 μL was used for LC-MS/MS analysis on a Q-Exactive mass spectrometer (Thermo Fisher Scientific) equipped with an Ultimate 3000 HPLC (Thermo Fisher Scientific). Peptides were separated by reverse phase chromatography on a C18 column (1.7 μm bead size, 75 μm × 20 cm, 65°C) at a flow rate of 400 nL/min using solvent A (0.1% TFA in water) and solvent B (0.1% TFA in acetonitrile). LC separation was performed using a 50 min linear gradient of 5 to 30% solvent B followed by a 15 min linear gradient of 30 to 75% solvent B. Survey scans were recorded over a 200−2000 m/z range (70,000 resolutions at 200 m/z, AGC target 1 × 10^6^, 75 ms maximum). MS/MS was performed in DDA mode with HCD fragmentation (30 normalized collision energy) on the ten most intense precursor ions (17,500 resolutions at 200 m/z, AGC target 5 × 10^4^, 120 ms maximum, dynamic exclusion 15 s). Peak integration and peptide data analysis were performed using PEAKS (v 8.5) software (Bioinformatics Solutions Inc.) exactly as outlined previously (69). The frequency of amino acids surrounding the cleavage sites was visualized using iceLogo software (70). The raw and processed mass spectrometry data files can be obtained through https://massive.ucsd.edu under the data set identifier number MSV000089434.

### Sessile Cell Adherence.

Cells were resuspended in mDixon broth and sieved through 45-μM cell strainer to remove cell clumps. Cell density was diluted to 1 OD_A600_/100 μL in each well in flat bottom polystyrene microtiter plate (Nunc, Thermo Fisher Scientific). Plates were sealed with adhesive film (4titude) and incubated at 32°C in a static incubator for 24-h. Plates were gently agitated at 60 rpm on orbital shaker (Major Science) for 1 min and washed with 150 μL of sterile PBS to remove non-adherent cells. Washing steps including shaking at 60 rpm at each interval were repeated three times before equal volume of XTT (2,3-bis-(2-methoxy-4-nitro-5-sulfophenyl)-2H-tetrazolium-5-carboxanilide) developer, and electron mediator solution (Abcam) in PBS was added to each well. Plates were incubated in the dark, 32°C for 2-h after which absorbance was measured at 450 nm with 75 μL of supernatant.

### Cell Surface Hydrophobicity Assay.

Fungal cell surface hydrophobicity was assessed using the microbial adhesion to hydrocarbons (MATH) method (42). Cells were washed twice with PBS and resuspended in 2.6 mL PBS to final OD_A600_ 0.5 (A0). Cell suspension was overlaid with 0.4 mL of n-hexadecane (Sigma-Aldrich), vortex for 2 min, and allowed to stand for 15 min at room temperature to ensure complete phase separation. OD_A600_ of the aqueous phase was measured (A1), and the percentage of hydrophobicity properties was calculated as hydrophobicity (%): [1−(A1/A0)] *100.

### Identification of EPS Protein with Proteome-Wide Mass Spectrometry.

48-h sessile phase cells of both WT and *mfsap1*Δ shake flasks cultures were gently washed twice with 20 mL of cold sterile water to remove loose/non-adherent cells. Sessile cells were collected in 10 mL cold sterile water containing protease inhibitors (complete ULTRA, Merck) and sonicated at 30% amplitude on 550 Sonic Dismembrator (Fisher Scientific) for 5 min while kept on ice. Cells were centrifuged thrice at 3,600 g for 15 min, 4°C and each time the supernatants were transferred to fresh tubes. Supernatants were further clarified with 0.22-μm filter (Minisart) and kept overnight at −80°C before lyophilization. Lyophilized EPS was resuspended in 2.5 mL buffer containing 25 mM sodium phosphate with 150 mM sodium chloride, pH 6.8, and applied to desalting PD-10 columns (GE Healthcare). Equivalent concentrations of soluble proteins were resolved on NuPAGE gels, and gel bands were excised and subjected to reduction and denaturation with 20 mM TCEP for 20 min at 55°C followed by alkylation with 55 mM CAA at RT for 30 min. Proteins were digested with trypsin overnight at 37°C, and samples were subsequently collected and dried with centrifugal vacuum evaporator. The digested peptides were solubilized in 0.5% acetic acid, desalted, and filtered using C18 and C8 StageTips, respectively. The peptides were analyzed using the Easy-nLC system coupled with an Orbitrap Fusion or Fusion Lumos Tribrid mass spectrometer (Thermo Fisher Scientific) and separated on a 50 cm × 75 µm Easy-Spray column. The LC-MS/MS parameters for Fusion: peptides were separated over a 70 min gradient, using mobile phase A (0.1% formic acid in water) and mobile phase B (0.1% formic acid in 99% acetonitrile), and eluted at a constant flow rate of 300 nL/min using 2 to 23% acetonitrile over 45 min, ramped to 50% over 15 min, then to 90% over 5 min and held for 5 min. Acquisition parameters: data-dependent acquisition (DDA) with survey scan of 60,000 resolution, AGC target of 4 × 10^5^, and maximum injection time (IT) of 50 ms; MS/MS collision induced dissociation in ion trap, AGC target of 1.0e4, and maximum IT of 50 ms; collision energy 35%, isolation window 1.2 m/z. The LC-MS/MS parameters for Fusion Lumos were as above with the following modifications: The gradient was 2 to 27% acetonitrile for the first 45 min, the maximum IT for MS was 100 ms, and the target AGC for the MS/MS was 1.5 × 10^4^. Peak lists were generated in Proteome Discoverer 2.3 (Thermo Scientific) using Mascot 2.6.1 (Matrix Science) and concatenated forward/decoy protein sequences obtained from protein sequences assigned using FUNAnnotate with (BioProject: PRJNA286710) *M. furfur* CBS14141 sequenced genome (71). Search parameters: MS precursor mass tolerance 10 ppm, MS/MS fragment mass tolerance 0.8 Da, three missed cleavages; static modifications: Carbamidomethyl (C); variable modifications: Acetyl (Protein N-term), Deamidated (NQ), and Oxidation (M). FDR estimation with two levels: Strict = FDR 1%, Medium = FDR 5%. Precursor mass peak (MS1) intensities were quantified by label-free quantification (LFQ) using the Minora feature detector. The raw spectra and search data were uploaded to the Jpost repository with the following accession numbers: JPST001565 (jPOST) and PXD033336 (ProteomeXchange). The detected proteins were functionally shortlisted to include secreted proteins with predicted signal sequence (SignalP5.0b, Linux x86_64). Protein FC of >1.5 was used to determine protein abundance by quantifying *mfsap1*Δ sample against WT reference and peptides were screened for predicted preferred cleavage sites (from tetradecapeptides protease substrate profiling data).

### Mfsap1 Protein-Based Complementation Assay.

WT and *mfsap1*Δ cells were inoculated at OD_A600_ 0.5 in 8 mL mDixon. At 0-h, 15% of cell-free 48-h CM obtained from *M. furfur* CBS14141 WT, *mfsap1*Δ, and 5.22 µg of recombinant Mfsap1 protein (equivalent to 15% of native Mfsap1 concentration in WT culture) were added to respective *mfsap1*Δ flasks. Similarly, 15% of clarified 48-h *mfsap1*Δ-CM was added to WT flasks to assess for potential inhibitory effects. Recovery of planktonic cells was monitored using OD_A600_ absorbance measurement over 48-h.

### Microbial Colonization on 3D RHE.

Monolayer of immortalized N/TERT keratinocytes was first cultured in CnT-PR epithelial culture medium (CellnTec) until 80% confluency. N/TERT-RHEs were generated by seeding 1 × 10^5^ N/TERT cells in 400 μL CnT-PR medium into 24-well polycarbonate membrane inserts (Nunc) for 48-h. After which, cultures were switched to CnT-prime 3D medium (CellnTec) for 24-h before subsequent air-lift incubation for 10 d to promote cell differentiation and stratification. For microbial colonization, 1 × 10^7^ cells/100 μL of WT and *mfsap1*Δ cell suspension in serum-free RPMI-1640 (Gibco) were applied to individual RHE, respectively. The co-cultures were maintained at 34°C, 5% CO_2_ for 6-h and the yeast RPMI-1640 diluent were removed by blotting with UV-radiated filter paper (Whatman) and allowed to further incubate for a total of 24-h. The colonized RHEs were gently washed thrice with 200 μL PBS to remove non-adherent yeast cells and fixed in 4% paraformaldehyde. The N/TERT skin equivalents were then embedded in paraffin and stained with hematoxylin and eosin using standard protocol.

### Multiplex Microbead-Based Immunoassay, Luminex assay.

Supernatants were collected for Luminex analysis using the MILLIPLEX MAP Human Cytokine/Chemokine Magnetic Bead Customized 7-plex Panel (Merck Millipore) to measure the following targets: IL-1α, IL-1β, IL-1RA, IL-6, IL-8, IL-10, and TNF-α. Harvested supernatants and standards were incubated with fluorescent-coded magnetic beads pre-coated with respective antibodies in a black 96-well clear-bottom plate overnight at 4°C. After incubation, plates were washed five times with wash buffer (PBS with 1% BSA (Capricorn Scientific) and 0.05% Tween-20 (Promega)). Sample-antibody-bead complexes were incubated with biotinylated detection antibodies for 1-h, and subsequently, Streptavidin-PE was added and incubated for another 30 min. Plates were washed five times again, before sample-antibody-bead complexes were re-suspended in sheath fluid for acquisition on the FLEXMAP® 3D (Luminex) using xPONENT® 4.0 (Luminex) software. Data analysis was done on Bio-Plex Manager^TM^ 6.1.1 (Bio-Rad). Standard curves were generated with a five-parameter logistic algorithm, reporting values for both mean florescence intensity (MFI) and concentration data.

### In Vivo Epicutaneous Infection of Mice.

All mouse experiments in this study were conducted in strict accordance with the guidelines of the Swiss Animals Protection Law and were performed under the protocols approved by the veterinary office of the Canton Zurich, Switzerland (license number 168/2018). All efforts were made to minimize suffering and ensure the highest ethical and humane standards according to the 3R principles (72). WT mice were purchased by Janvier Elevage (France) and maintained at the Laboratory Animal Science Center of the University of Zurich, Zurich, Switzerland. Mice were used at 6–12 wk in sex- and age-matched groups. Epicutaneous infection of the mouse ear skin was performed as described previously (44, 73). Briefly, *Malassezia* strains were grown in mDixon medium at 30°C, 180 rpm for 2–3 d. Cells were washed in PBS and suspended in native olive oil at a density of 10 OD_A600_/mL. 100 µL suspension (corresponding to 1 OD_A600_) of yeast cells was applied topically onto the dorsal ear skin that was previously disrupted by mild tape stripping while mice were anaesthetized. Ear thickness was measured prior and during the course of infection using the Oditest S0247 0–5-mm measurement device (Kroeplin). For determining the fungal loads in the skin, tissue was transferred in water supplemented with 0.05% Nonidet P40 (AxonLab), homogenized and plated on mDixon agar, and incubated at 30°C for 3–4 d.

### Histology.

Mouse tissue was fixed in 4% PBS-buffered paraformaldehyde overnight and embedded in paraffin. Sagittal sections (9 µm) were stained with periodic acidic Schiff (PAS) reagent and counterstained with hematoxylin and mounted with Pertex (Biosystem, Switzerland) according to standard protocols. All images were acquired with a digital slide scanner (NanoZoomer 2.0-HT, Hamamatsu) and analyzed with NDP view2.

### Mouse RNA Extraction and Quantitative RT-PCR.

Isolation of total RNA from murine ear skin was carried out according to standard protocols using TRI reagent (Sigma-Aldrich). cDNA was generated by RevertAid reverse transcriptase (Thermo Fisher Scientific). Quantitative PCR was performed using SYBR green (Roche) and QuantStudio 7 Flex (Life Technologies) instrument. The primers used for qPCR are listed in *SI Appendix*, Table S7. All RT-qPCR assays were performed in duplicate, and the relative expression (rel. expr.) of each gene was determined after normalization to *Actb* transcript levels.

### Isolation of Murine Skin Cells and Flow Cytometry.

For digestion of total ear skin, mice ears were removed, cut into small pieces, and transferred into Hank’s medium (Ca^2+^ and Mg^2+^ free, Life Technology), supplemented with Liberase TM (0.15 mg/mL, Roche) and DNase I (0.12 mg/mL, Sigma-Aldrich) and incubated for 1h at 37°C. The cell suspension was filtered through a 70-µm cell strainer (Falcon) and rinsed with PBS supplemented with 5 mM EDTA (Life Technologies), 1% fetal calf serum and 0.02% NaN_3_. Single-cell suspensions were stained with antibodies directed against CD45 (clone 104), CD11b (clone M1/70), Ly6C (clone HK1.4), and Ly6G (clone 1A8). LIVE/DEAD Near IR stain (Life Technologies) was used for exclusion of dead cells. All staining steps were carried out on ice. Cells were acquired on a Spectral Analyzer SP6800 (Sony), and the data were analyzed with FlowJo software (FlowJo LLC). The gating of the flow cytometric data was performed according to the guidelines for the use of flow cytometry and cell sorting in immunological studies (74), including pre-gating on viable and single cells for analysis. Absolute cell numbers were calculated based on a defined number of counting beads (BD Bioscience, Calibrite Beads), which were added to the samples before flow cytometric acquisition.

## Supplementary Material

Appendix 01 (PDF)

Dataset S01 (XLSX)

## Data Availability

EPS mass spectrometry proteomics data have been deposited in jPOST and ProteomeXchange (JPST001565, PXD033336) and files can be obtained through https://repository.jpostdb.org/entry/JPST001565. All sequencing data were deposited under BioProject Accession ID: PRJNA658716. All study data are included in the article and/or *SI Appendix*.
