# Supplementary material for: The human pathobiont Malassezia furfur secreted protease Mfsap1 regulates cell dispersal and exacerbates skin inflammation
Source: Proc Natl Acad Sci U S A. 2022 Nov 29;119(49):e2212533119. doi: 10.1073/pnas.2212533119 (PMC9894114; doi:10.1073/pnas.2212533119)
Supplement: Supplementary file 1 — Appendix 01 (PDF) [file pnas.2212533119.sapp.pdf]

PNAS

Supporting Information for

The human pathobiont *Malassezia furfur* secreted protease Mfsap1 regulates cell dispersal and exacerbates skin inflammation

Joleen P.Z. Goh[1,2], Fiorella Ruchti[3], Si En Poh[4], Winston L.C. Koh[5,6], Kiat Yi Tan[7], Yan Ting Lim[7], Steven T.G. Thng[8,11], Radoslaw M. Sobota[7], Shawn S. Hoon[4], Chenxi Liu[9], Anthony J. O'Donoghue[9], Salomé LeibundGut-Landmann[3], Hazel H. Oon[8], Hao Li[4,10*] and Thomas L. Dawson, Jr[1,11,12*]

[1]A*STAR Skin Research Labs (A*SRL), Agency for Science, Technology and Research, Singapore, Singapore

[2]Lee Kong Chian School of Medicine, Nanyang Technological University, Singapore, Singapore

[3]Section of Immunology, Vetsuisse Faculty and Institute of Experimental Immunology, University of Zürich, Zürich, Switzerland

[4]Molecular Engineering Lab, Institute of Molecular and Cell Biology, Agency for Science, Technology and Research, Singapore, Singapore

[5]Institute of Bioengineering and Bioimaging, Agency for Science, Technology and Research, Singapore, Singapore

[6]Bioinformatics Institute, Agency for Science, Technology and Research, Singapore, Singapore

[7]Functional Proteomics Laboratory, Institute of Molecular and Cell Biology, Agency for Science, Technology and Research, Singapore, Singapore

[8]National Skin Centre, National Healthcare Group, Singapore, Singapore

[9]Skaggs School of Pharmacy and Pharmaceutical Sciences, University of California San Diego, La Jolla, California

[10]Department of Chemistry, National University of Singapore, Singapore, Singapore

[11]Skin Research Institute of Singapore, Agency for Science, Technology and Research, Singapore, Singapore

[12]Department of Drug Discovery, School of Pharmacy, Medical University of South Carolina, Charleston, SC, USA

***Correspondence:**
Both authors contributed equally to this manuscript.

Hao Li, **chmlihao@nus.edu.sg**
Thomas L. Dawson, Jr, **thomas_dawson@asrl.a-star.edu.sg**

This PDF file includes:
Figures S1 to S7
Tables S1 to S7
SI References

Other supporting materials for this manuscript include the following:
Software S1

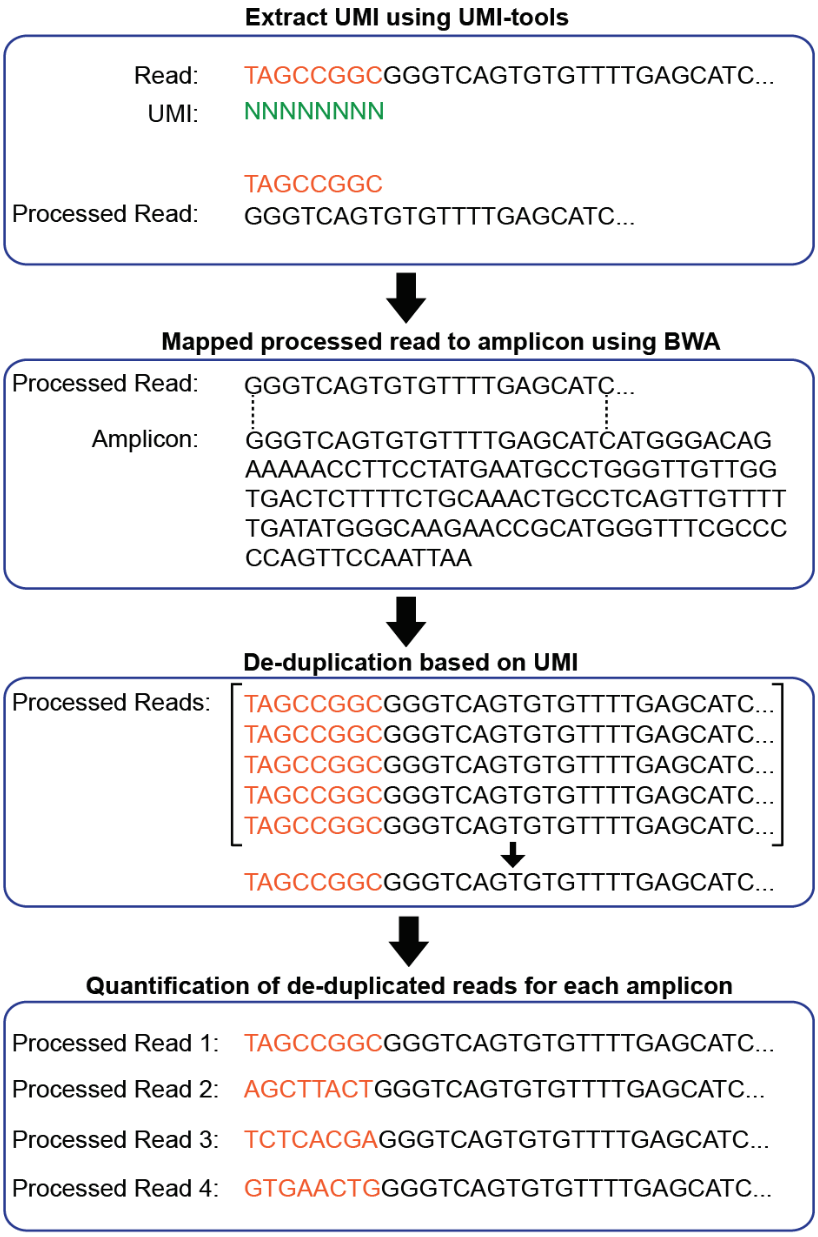

Figure S1. Schematic of the overall workflow involved in analysing the unique molecular identifiers (UMI) labelled transcripts for gene expression analysis.

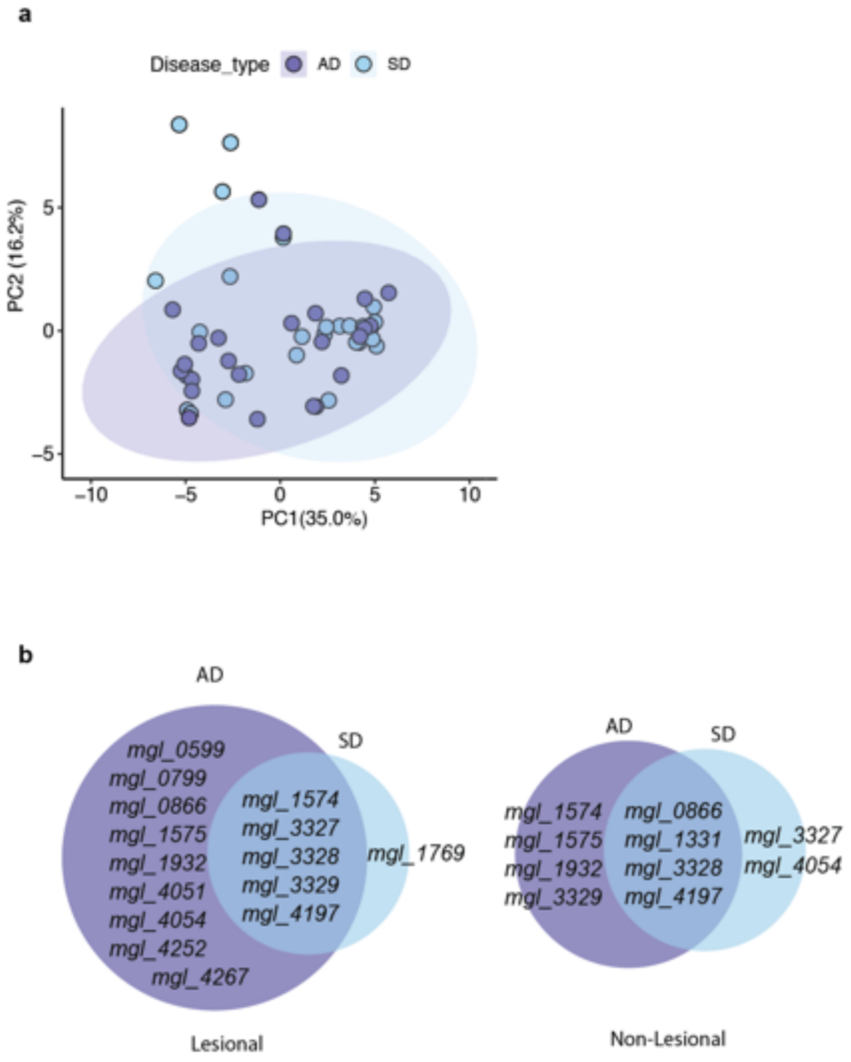

Figure S2. AD and SD patients show similar *M. globosa* secretory enzyme gene expression. **(a)** Principal component analysis plot of the 42 *M. globosa* genes analysed in this study for both the AD and SD subjects. Non-lesional (NL) and lesional (L) samples are both included. n = 25 for AD (n = 8 for NL, 17 for L); n = 26 for SD (n = 10 for NL, 16 for L). **(b)** List of differentially expressed genes for AD and SD subjects as compared to healthy subjects. Differential expression is determined by a threshold of > 1.5-fold change and p-value of <0.05.

>MfSAP1(FUN_000223) *Malassezia furfur* Secreted Aspartyl Protease 1
MQLSLKFVTGLLLATSAVFAAQDDIEVNLHRRNSFITAGNQLNVEAFAKHLLNVNNKYKDALENFKRNMGHDHPL
LSILLNGIKR**NNGGQGSLPLTDVQSELLWSGDVTFGGQTFGMDFDTGSSDSLANPGAYNPRKSSTSKNTWSSFSA**
AYGDGTQARGLVYTDDFEIAGLKAKNVAIGHSFTKFIEDQEPAQGIAGMAFPSIQTFPKQYKPFFESLKEQKAVK
SGVFQFTLKAGKGSTLTLGGIDSSKYKGKVTYVDVDPSQGFWITDAKVNGHGIKAIIDSGSTIITGPTSQVRSVV
QNIKGMNAFNQDGTLMYTYDCNSTPNVTISIGGTPFKLGKNQLSYGRANGQCVLPIAGQDGLPLNAWIVGDTFFQ
SVSVIFDTDKNRMGFATQA

Protein sequence of full length MfSAP1. The predicated signal peptide (using SignalP 5.0) is highlighted in green, with cleavage site between position 20 and 21: VFA-AQ. Pro-peptide is shown in red, and the mature protease sequence is shown in bold.

>MfSAP2 (FUN_000222) *Malassezia furfur* Secreted Aspartyl Protease 2
MRITIPLLSLFLLAASNVFAGPVASKYETGIPISLQRRARFVSANGHVNLVALTRHYKGLDSKYSKCLDNYRRNT
GHKHPLQAHRHKKREHPSKHGHGHPNRSRDKSRKDGFGGIDGGMGGGSCPDLGGPDGGMPGGMGGMPGGGSSPGS
PSSSGGGSGGSPSSSGGGSGSSPNGSGGGSGSGSGSGSGSGSGSGSGSGSGSGSGSGSGSGSGSGSDGGSDGD
SVSLTDVGNQQLWAGDITFGGQTFGIDFDTGSADTLANPSAYDPRKSDTAQNTNQPFQTSYGDGTAAQGTIWTDS
FQIAGFSAENVAIGVADQDFITPDQQPSQ*GISG*MSFPSLQAFPKEYPPFFESLRQQKVVNQGVFQFTLKAGEGSE
LHLGGVDESKAQGDFSWASVDPSQGFWVTDAAINGQQIKAIIDSGSTIFSGPTDEVRSLMQTIPGAMPSPQNGAT
AYLYDCDKSPDVTITVAGLDVQLSRDQMRFGQTQGKCMLPIMGLDGIPLNAWIVGDTLFQATTIVFDMDENRMGF
ALQA

Protein sequence of full length MfSAP2. The predicated signal peptide (using SignalP 5.0) is highlighted in green, with cleavage site between position 20 and 21: VFA-GP. Pro-peptide and mature protease sequences are undetermined. *Putative motif sequence/s are italicised and underlined.

>MfSAP3 (FUN_001776) *Malassezia furfur* Secreted Aspartyl Protease 3
MANSLVRWVALLVPLLTLVCASNVTHVALTPPATPVSAASYAEAVAWVDAHDDYVHRKYTRYDRRSLQPAPLLNY
RHDGMWTATIDVGTPPKEHQVVVDTGSADLWLSSRSYMPKKSRTYKTLNKPFSLTYNVGSAQGYAASDRVAIGGV
VADPQYFGVVNHSEHLTLPTNVTGILGLALPDLSVLHAQPFWAASNLSPRVMGVYLKRDPHPGQGANNKAGGVLT
LGGANTSLYEGDIDYIQVTDKRHWQVPLNGLAVEGAHIHLPRDTQAFFDTGTALIGGPSDVVHRVYTKIPGSKPM
PQNKGHFTYPCAAQPNISFSFGQRKYYLNNADFRAMVIQTDNDA*IDGE*RCMDEQ

Protein sequence of full length MfSAP3. The predicated signal peptide (using SignalP 5.0) is highlighted in green, with cleavage site between position 21 and 22: VCA-SN. Pro-peptide and mature protease sequences are undetermined. *Putative motif sequence/s are italicised and underlined.

>MfSAP4 (FUN_003258) *Malassezia furfur* Secreted Aspartyl Protease 4
MLTGLVGRAVCAAVLLCQFANAEPTPTPSAHRAEGTGIQVPIRSNSEALHPRNGNLEGDAFVQWMDRERNALNAK
YNSTRKHKNAKKHARQLVGIGNYGQNSFYFMPIGIGTPSTTVNVLMDTGSSDFWIADASCSELTGCSESMTLYDP
SKSSTFNSSDRTFLTPYGDGTNTVSGKLGADDVTMAEYQVDGLTFGRVSQLTGSTIQPPASGLMGMGFESLSSSE
STPFWEVVALQGKVKDPVFSFQLASADSSDSASKVVPGGVFSLGVLDDRQYTGDIAWVDLTEGYGSQGIGYWAIT
MDKLVANGETIELDQQNIVAVDTGTTLIGGPQSILRQIYSQIPGVRSAPSYLLGGSGYYMYPCTQPFTLKMTFGG
KEFTLDNENLNLGRLSSRSNMCISSLFDAPQSQNSAMPAWILGDTFLRTVFSVYSWNPERVGFASLPSGGASTLP
MTSITSGETFSDASTSLAGGGSVTSSALLTSSSSVHSRQTGLLGGNGLPTPSLVSVPTGFQSLASLRSYGLGGNQ
DNGVQVPYSVYLVLYSAVIATVTAAFVL

Protein sequence of full length MfSAP4. The predicated signal peptide (using SignalP 5.0) is highlighted in green, with cleavage site between position 22 and 23: ANA-EP. Pro-peptide and mature protease sequences are undetermined.

>MfSAP5 (FUN_003259) *Malassezia furfur* Secreted Aspartyl Protease 5
MRCLLLVLASLVASGIAAPSPTQSAAPTTSPGIRLPILVDRDLPHPRNKNLQGDALLQWMQRERTHVTNKYSKHG
NKNRGNNRNKMHARQAAPLGSVNLGGFYFSQIGLGTPEKTYNVVLDTGSADFWIASTSCSGCDNMNLFEPSDSST
YQSSEQQFEVPYGQGGVRGSMGADNVSLAGYKISGLNFGIATQLASGTIQPPASGIMGMGFESLSSSGSTPFWQV
VAIEGKLKDPVFSFELTDNTDYSSAGQVTAGGVFTLGNLDDQQYSGDITWIDLDSRYGSKGIGYWGIKMDALSVN
GQNINLGQHNLVAVDTGTTLIGAPESVVEAVYAQIPNAEPASSSTFGGQGYYVFPCSQKFEIAFTFGGKAFTLNQ
DDINIGQVDMGRETCGGALFVVDSPAGSATPGWILGDTFLSKVYSVYSWQPQRVGFASLPSNGPRTLALTETSGG
NTVASAAASNGGGGGISEPMSRSRMQLTGVVGGEGLPTPTLIDVPSGMQKLSAPSSLPTGWNGGVGGGVSQVTVR
NSAQWASLTSELGGDGGGIFSIFGGGNSAQSRTGGVPVMLISLVAATLVACFAL

Protein sequence of full length MfSAP5. The predicated signal peptide (using SignalP 5.0) is highlighted in green, with cleavage site between position 17 and 18: GIA-AP. Pro-peptide and mature protease sequences are undetermined.

(I) Asp-Thr-Gly: D-T-G
(II) Gly-Hydrophobic-Hydrophobic-Gly
: G-(A/I/L/M/F/W/Y/V)-(A/I/L/M/F/W/Y/V)- G
(III) Asp-Thr/Ser-Gly: D-T/S-G
(IV) Ile-Hydrophobic-Gly-Asp/Gln/Asn
: I-(A/I/L/M/F/W/Y/V)-G-(D/Q/N)

Figure S3. Protein sequence of full length MFSAP1-5 including predicted signal peptide using SignalP 5.0 and the four major aspartyl protease active sites highlighted in yellow.

Catalogue No.	Sequence
AMYD-103	Mca-VNLDAEF-K(Dnp)-NH2
AMYD-105	Mca-SEVNLDAE-Dap(Dnp)-NH2
AMYD-108	Mca-SEVNLDAEF-K(DNP)-NH2
AMYD-109	Mca-SEVNLDAEFR-K(DNP)-R-R-NH2
AMYD-110	Mca-SEVKMDAEFR-K(DNP)-RR-NH2
AMYD-111	Mca-RPPGFSAFK-(Dnp)-NH2
AMYD-112	Mca-HQKLVFFA-K(DNP)-NH2
AMYD-114	Mca-EVKMDAEF-K(DNP)-NH2
MMPS-009	Mca-PLGL-Dap(Dnp)-AR-NH2
MMPS-016	Mca-P-Cha-G-Nva-HA-Dap(DNP)-NH2
MMPS-024	Mca-RPKPYA-Nva-WM-K(Dnp)-NH2
MMPS-026	Mca-RPKPVE-Nva-WRK(DNP)-NH2
MMPS-029	Mca-KPLGL-Dap(Dnp)-AR-NH2
CASP-027	Mca-YVADAP-K(DNP)-NH2
CASP-028	Mca-VDQVDGW-K(Dnp)-NH2
CASP-059	Mca-DEVDAP-K(Dnp)-NH2
CASP-060	Mca-VDQMDGWK-(DNP)-NH2
CASP-068	Mca-LEVDGWK(DNP)-NH2
SUBS-017	Mca-GKPILFFRL-K(Dnp)-DArg-NH2

Figure S4. Synthetic FRET substrate peptide sequences (CPC Scientific). Mca: (7-methoxycoumarin-4-yl) acetyl, DNP: 2,4-dinitrophenol, Dap: 2,3-Diaminopropionic acid, Nva: Norvaline, r: D-arginine.

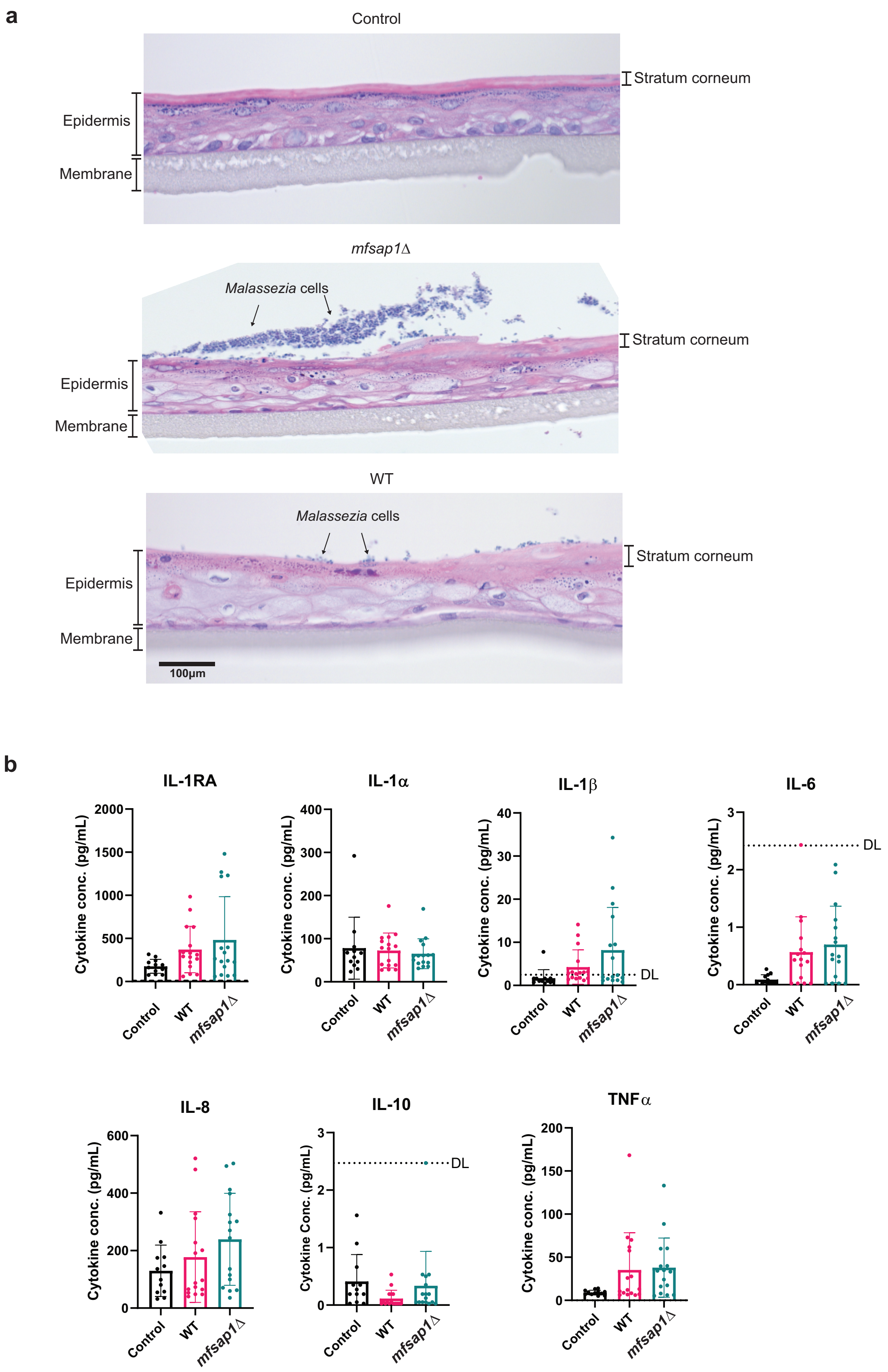

Figure S5. (a) Representative histological sections of reconstructed human epidermis (RHE) stained with hematoxylin and eosin. Scale bar = 100 µm. **(b)** Cytokine profile of culture medium supernatant collected from control and infected RHE. DL, detection limit.

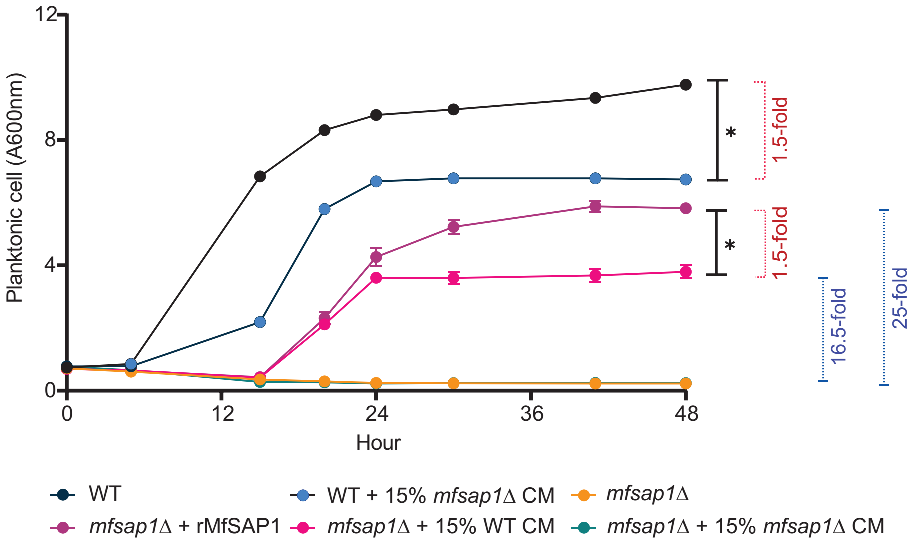

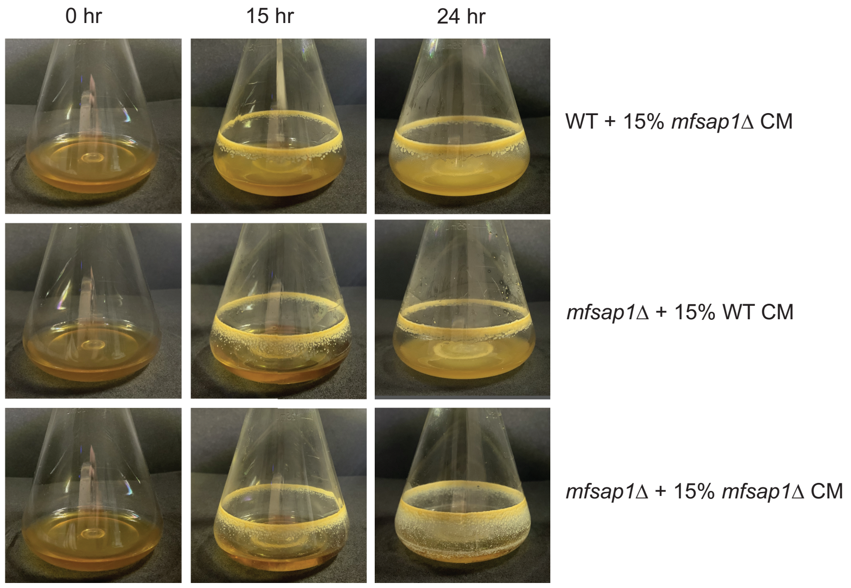

Figure S6. Protein-based complementation assay. *mfsap1*Δ liquid cultures were supplemented with 15% *M. furfur* WT conditioned medium (CM), the equivalent amount of recombinant Mfsap1 protein (rMfsap1, 5.22 μg), or 15% of *mfsap1*Δ conditioned medium as control.

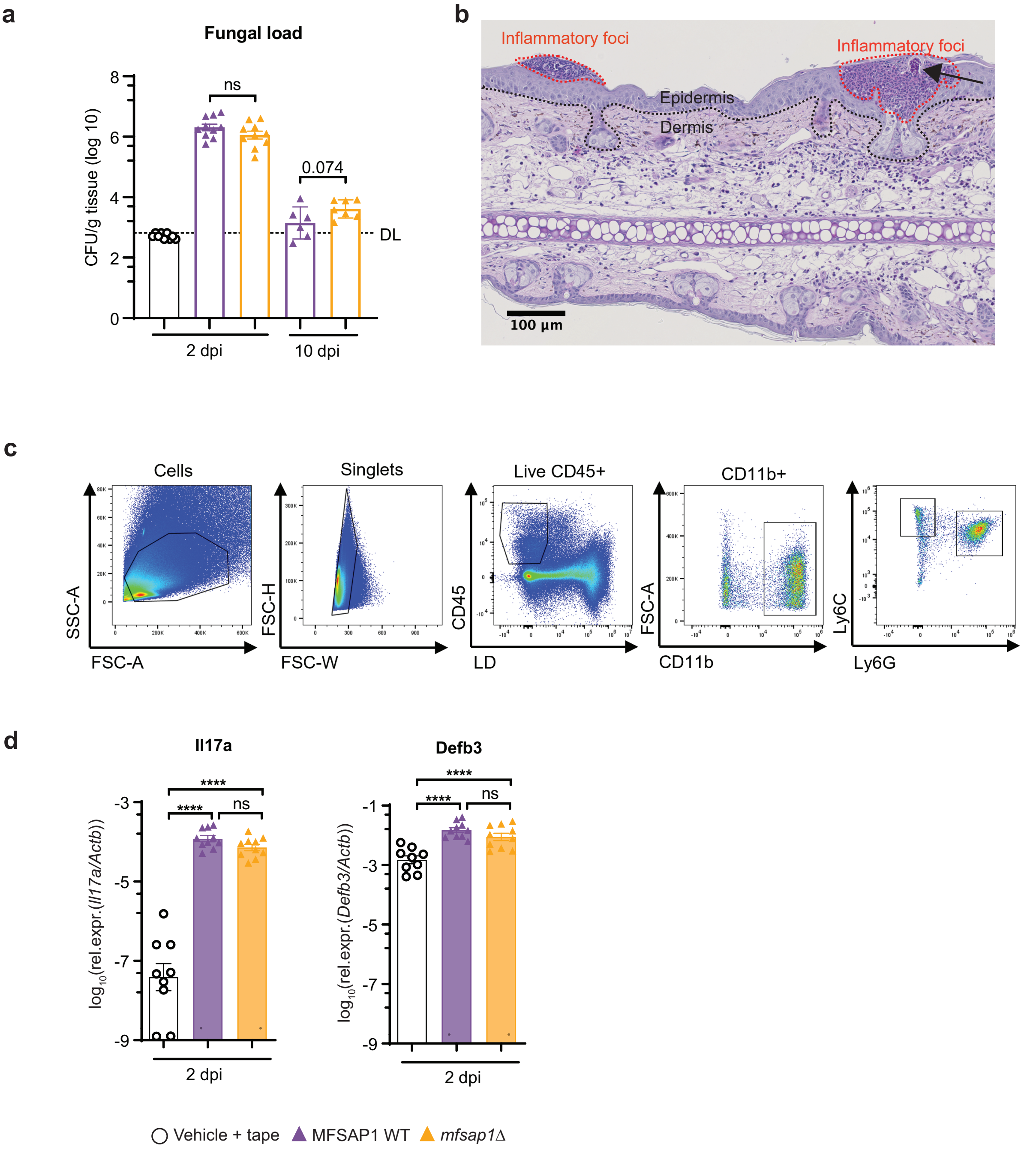

Figure S7. C57BL/6 wildtype mice were tape stripped and epicutaneously infected with MFSAP1 WT or *mfsap1*Δ. **(a)** Colony forming units (CFU) taken from mouse ear skin on 2 and 10 dpi. **(b)** Magnification of the histological section shown in Figure 4D from the MFSAP1 WT group. Red outline highlights zones highly infiltrated by myeloid cells (inflammatory foci) in the epidermis; the black arrow indicates an accumulation of *M. furfur* cells within an inflamed hair follicle. Scale bar = 100 µm. **(c)** Representative flow cytometry plots showing the gating strategy for detection of CD45+ leukocytes,

CD11b$^+$ Ly6C^{hi} Ly6G$^-$ monocytes and CD11b$^+$ Ly6G$^+$ neutrophils in ear skin. **(d)** IL-17a and β-defensin 3 gene expression in mouse ear skin at 2 dpi.

Data are pooled from two- or three independent experiments for the 2 dpi and 10 dpi timepoints, respectively, with 3-4 mice per group each. Statistics were calculated using one-way ANOVA with Šídák's multiple comparisons test. ****$p<0.0001$.

Table S1. List of *M. globosa* gene targets and specific primer used for targeted RNA-seq.

**Gene Locus**	**Enzyme**	**Illumina P5 adaptor+8N barcode+forward primer**
mgl__0285	Protease	CACGACGCTCTTCCGATCTNNNNNNNNGGTGCTGGCAATTTAGCCTT
mgl__0534	Protease	CACGACGCTCTTCCGATCTNNNNNNNNTCCTTGAGCACGTTTCGCTC
mgl__0599	Protease	CACGACGCTCTTCCGATCTNNNNNNNNCATGGTGGAAACACTCCTGG
mgl__0815	Protease	CACGACGCTCTTCCGATCTNNNNNNNNGGTTTCCAAAGGCCACCGA
mgl__0866	Protease	CACGACGCTCTTCCGATCTNNNNNNNNTGGACACGCGTAGGTCAAC
mgl__1260	Protease	CACGACGCTCTTCCGATCTNNNNNNNNCTATGGACGGTGCTGACTGG
mgl__1932	Protease	CACGACGCTCTTCCGATCTNNNNNNNNGGGTCAGTGTGTTTTGAGCATC
mgl__2125	Protease	CACGACGCTCTTCCGATCTNNNNNNNNATGCCGCCTTCCGATGAATG
mgl__2506	Protease	CACGACGCTCTTCCGATCTNNNNNNNNGCGCTTGTGAATAACCGCCT
mgl__3195	Protease	CACGACGCTCTTCCGATCTNNNNNNNNAGGTACCTGTATTTCGCCGTT
mgl__3327	Protease	CACGACGCTCTTCCGATCTNNNNNNNNGTCAGCATCAACGGCTTCAA
mgl__3328	Protease	CACGACGCTCTTCCGATCTNNNNNNNNAAAAGATGCTATGGTTGCGGG
mgl__3329	Protease	CACGACGCTCTTCCGATCTNNNNNNNNGCGCTTACTCTGGCAACTGA
mgl__3330	Protease	CACGACGCTCTTCCGATCTNNNNNNNNGTTGGTGACGGAAAGTGCAT
mgl__3331	Protease	CACGACGCTCTTCCGATCTNNNNNNNNATGAAGAAGTACGACCTTGGCA
mgl__3565	Protease	CACGACGCTCTTCCGATCTNNNNNNNNCATGACTAGGGAGCACGACA
mgl__3647	Protease	CACGACGCTCTTCCGATCTNNNNNNNNTCGAGCAATTCTTCGCCCA
mgl__3649	Protease	CACGACGCTCTTCCGATCTNNNNNNNNACATCCGGCAGTTCTTCGAG
mgl__4053	Protease	CACGACGCTCTTCCGATCTNNNNNNNNTCGTGATCAGGTTTCCCGTG
mgl__4243	Protease	CACGACGCTCTTCCGATCTNNNNNNNNCTCGACTTTGACAACCACCG
mgl__4267	Protease	CACGACGCTCTTCCGATCTNNNNNNNNGAGGCAAGCGTTTCACGATG
mgl__0797	Lipase	CACGACGCTCTTCCGATCTNNNNNNNNTTGGATCTACCCAGGCAACAG
mgl__0798	Lipase	CACGACGCTCTTCCGATCTNNNNNNNNCTGGAGGCTTTACCCTGGAC
mgl__0799	Lipase	CACGACGCTCTTCCGATCTNNNNNNNNGAATGCTCACTGTAGCCCGT
mgl__0800	Lipase	CACGACGCTCTTCCGATCTNNNNNNNNACCCGGGCAATTCAACTAAT
mgl__1331	Lipase	CACGACGCTCTTCCGATCTNNNNNNNNACCATACCGGTGTATATTTCCGA
mgl__3878	Lipase	CACGACGCTCTTCCGATCTNNNNNNNNCTACCCTGGACAAGAGAACCAC
mgl__1769	Lipase	CACGACGCTCTTCCGATCTNNNNNNNNTATGGCACACCGCATCACA
mgl__1311	Lipase	CACGACGCTCTTCCGATCTNNNNNNNNTTGACGTGGGCATCAACACTA
mgl__3507	Lipase	CACGACGCTCTTCCGATCTNNNNNNNNGAGTGCTCACGCTAAGTACC
mgl__4051	Lipase	CACGACGCTCTTCCGATCTNNNNNNNNTTCAATGGTAAGCAGTGGGGC
mgl__4052	Lipase	CACGACGCTCTTCCGATCTNNNNNNNNTCCTTTATGGCGACCCGACA
mgl__4054	Lipase	CACGACGCTCTTCCGATCTNNNNNNNNAGAAATCAAGAACCACGACGCG
mgl__4197	Lipase	CACGACGCTCTTCCGATCTNNNNNNNNGCCGAGAAATCAAGAACTACGACGTT
mgl__1067	Phospholipase C	CACGACGCTCTTCCGATCTNNNNNNNNACGACCAAGCTGCTGACTTT
mgl__3075	Phospholipase C	CACGACGCTCTTCCGATCTNNNNNNNNATCACGTCGTCTTTGGACCT
mgl__3076	Phospholipase C	CACGACGCTCTTCCGATCTNNNNNNNNTCTTGGAAATGGCGCTGGAT
mgl__3077	Phospholipase C	CACGACGCTCTTCCGATCTNNNNNNNNTGGGCAATGGTGCTGGATAC
mgl__3326	Phospholipase C	CACGACGCTCTTCCGATCTNNNNNNNNAAGAAGGACGATGGCGTTGT

mgl__4252	Phospholipase C	CACGACGCTCTTCCGATCTNNNNNNNNNCTGACGGACTCTCTGAAGCTC
mgl__1573	Sphingomyelinase	CACGACGCTCTTCCGATCTNNNNNNNNNCTCGTCACGACCTTTGCTCT
mgl__1574	Sphingomyelinase	CACGACGCTCTTCCGATCTNNNNNNNNNCCTTACCGACGAGATGGAGC
mgl__1575	Sphingomyelinase	CACGACGCTCTTCCGATCTNNNNNNNNNGAGGCACGTCCTGAACTGAT
mgl__3568	Sphingomyelinase	CACGACGCTCTTCCGATCTNNNNNNNNNTTGGGCTTCAATCTCCGATGA
mgl__1986	Housekeep	CACGACGCTCTTCCGATCTNNNNNNNNNAGATCACGGCTCTTGCTCCTA
mgl__0961	Housekeep	CACGACGCTCTTCCGATCTNNNNNNNNNATGAGGTTGTGTCGCAGGAC
mgl__1134	Housekeep	CACGACGCTCTTCCGATCTNNNNNNNNNCCTGCGACGTCAAAGGATGG

Table S2. Healthy volunteer recruitment characteristics.

Characteristics	Healthy volunteers
Mean age (SD)	28 (4)
**Gender**	
Male	16
Female	29
**Ethnicity**	
Chinese	36
Indian	3
Caucasian	4
Korean	1
Philipino	1

Table S3. Atopic Dermatitis Patient Recruitment Clinical Characteristics.

No.	Number	Ethnicity	Age	Gender	Current Treatment and Medications	SCORAD	Control Sites	Diseased Sites	Control site pass sequencing	Disease site pass sequencing
1	SM05	Chinese	22	Male	Ciclosporin	43.2	Frontal scalp, Neck	Occiput,Glabella	No	Yes
2	SM09	Malay	27	Male	None	64.5	Right Index Finger, Left Middle Finger	Frontal scalp , Glabella	No	Yes
3	SM10	Chinese	36	Male	None	43.8	Left temple, Lower Back	Glabella, Upper Back	Yes	Yes
4	SM11	Chinese	25	Male	None	40.9	Left Cheek , Left Chest	Glabella, Right Chest	No	No
5	SM14	Chinese	28	Female	None	36.7	Left retroauricular crease, Left occiput	Right frontal scalp, Mid- frontal Scalp	No	No
6	SM20	Chinese	41	Male	UVA/UVB	37	Right and left retroauricular crease	Glabella, Right temporal scalp	No	Yes
7	SM22	Chinese	27	Female	None	29.1	Left and right volar forearm	Left and right Cheek	No	Yes
8	SM33	Chinese	27	Male	Mycophenolat e Mofetil	24.1	Right and left retroauricular crease	Left and Right Forehead	Yes	Yes
9	SM34	Chinese	40	Male	Methotrexate	31.7	Right Earlobe, Left Scalp	Right Parietal Scalp, Left Occiput	Yes	Yes
10	SM36	Chinese	51	Male	None	57	Right and Left Upper Back	Right and left Mid-Back	No	Yes
11	SM38	Chinese	46	Female	None	56	Right Scalp, Glabella	Right Frontal Scalp, Left Forehead	Yes	Yes
12	SM40	Chinese	55	Female	PUVA soak (hand/feet), NBUVB, Atorvastatin, Aspirin	58.5	Right and Left Scalp	Right Cheek, Left Upper Back	Yes	Yes

13	SM42	Chinese	36	Female	Cetirizine	44.6	Left Cheek, Right Scalp	Right Cheek,Left Forehead	Yes	Yes
14	SM43	Malay	30	Male	Ciclopsorin	54.5	Right Cheek, Frontal Scalp	Left Cheek, Left Occiput	No	Yes
15	SM44	Chinese	27	Male	Azathioprine, Carbimazole	57	Frontal scalp	Right Cheek,Occiput	Yes	Yes
16	SM47	Chinese	21	Male	Ciclopsorin, Cetirizine	38.1	Right and left retroauricular crease	Right Forehead ,Left Forehead	No	Yes
17	SM48	Chinese	54	Female	Rosuvastin	46	Left scalp, Right retroauricular crease	Frontal scalp, Occiput	No	Yes
18	SM49	Chinese	25	Female	Cetirizine, Hydroxyzine	36	Right retroauricular crease, Right lower cheek	Left and Right Cheek	No	Yes
19	SM50	Malay	25	Male	Protopic, Betamethason e, UVB, Hydroxyzine	43.5	Right and left retroauricular crease	Left forehead, Left cheek	Yes	Yes
		**Mean age**	33.8					**Total**	8	17

Table S4. Seborrheic Dermatitis Patient Recruitment Clinical Characteristics.

No.	Number	Race	Age	Gender	Current Treatment and Medications	CSES	ASFS	Scalp IGA	Facial IGA	Control Sites	Diseased Sites	Control site pass sequencing	Disease site pass sequencing
1	SM07	Indian	24	Male	None	24	20	3	3	Occiput, Right Jaw	Right Cheek, Left Frontal Scalp	No	Yes
2	SM08	Chinese	29	Male	None	0	4	1	1	Right Back, Left retroauricular crease	Left Back, Right retroauricular crease	Yes	Yes
3	SM12	Chinese	31	Male	None	0	0	0	2	Right and Left retroauricular crease	Right and Left Cheek	No	Yes
4	SM15	Chinese	25	Female	None	16	24	3	0	Left retroauricular crease, Right frontal Scalp	Right and Left Occiput	Yes	Yes
5	SM16	Myanmar	34	Female	None	14	48	3	0	Right Cheek, Leftretroauricular crease	Right and Left Occiput	Yes	Yes
6	SM23	Caucasian	51	Male	Selegiline	16	36	3	2	Right and Left retroauricular crease	Glabella, Right frontal scalp	Yes	Yes
7	SM30	Others	30	Male	none	16	16	2	2	Right Occiput, Left Occiput	Right and left frontal scalp	No	Yes
8	SM31	Chinese	25	Female	none	8	8	1	1	Left retroauricular crease, Left Scalp	Right retroauricular crease , Right occiput	No	Yes
9	SM37	Malay	26	Female	none	4	4	2	2	Right Cheek, Left Cheek	Right alar crease, Left Frontal Scalp	Yes	Yes

10	SM41	Others	32	Female	none	8	8	2	2	Left frontal scalp, Left cheek	Right frontal scalp, Right cheek	Yes	Yes
11	SM52	Filipino	40	Male	None	12	12	2	2	Left cheek, Left retroauricular crease	Right cheek, Right frontal scalp	Yes	Yes
12	SM53	Indonesian	37	Male	None	16	40	3	1	Right and Left retroauricular crease	Left and right frontal scalp	Yes	Yes
13	SM54	Chinese	25	Male	None	21	6	2	1	Left cheek, right retroauricular crease	Right alar crease, Left occiput	No	Yes
14	SM55	Chinese	25	Male	Ivermectin cream 1%, brimonidine gel 0.33%	20	20	2	2	Left retroauricular crease, Glabella	Left Occiput, Right Occiput	Yes	Yes
15	SM56	Chinese	37	Male	None	0	4	1	3	Left Cheek , Right Cheek	Right alar crease, Right Glabella	Yes	No
16	SM57	Chinese	25	Male	None	1	4	1	2	Left retroauricular crease, Right Cheek	Left side cheek, Right Preauricular	No	Yes
17	SM59	Chinese	32	Female	None	8	6	2	2	Left retroauricular crease, Right Cheek	Left upper ear, Right glabella	No	Yes
		**Mean age**	31.1								**Total**	10	16

* CSES = Clinical Scalp Erythema Score (scale of 0-80)
* ASFS = Adherent Scalp Flaking Severity (scale of 0-80)
* IGA = Investigator Global Assessment (scale of 0-4)

Table S5. List of *M. furfur* SAP targets and specific qPCR primers.

Annotation	Gene	Forward Primer, 5' to 3'	Reverse Primer, 5' to 3'	Amplicon size (bp)
	CBS 14141 actin	CGGCAACATTGTCATGTCGG	GCAAGGATCGAACCACCGAT	159
FUN_000223	MfSAP1	TCGGTCACTCGTTCACCAAG	GAGGCTCTCAAAGAACGGCT	118
FUN_000222	MfSAP2	ACAGACGTTTGGCATCGACT	CGAAGTCTGGAATGGCTGGT	119
FUN_001776	MfSAP3	ATACGTCGCTGTATGAGGGC	TTGGGTATCACGGGGGAGAT	122
FUN_003258	MfSAP4	AAGAATGCGAAGAAGCACGC	CAGCACATTCACGGTTGTCG	144
FUN_003259	MfSAP5	CTCCTACTCGTGCTGGCTTC	TGCATCCACTGCAATAACGC	173

Table S6. List of MFSAP1 primers used in gene deletion construct.

Primer	Forward Primer, 5' to 3'	Reverse Primer, 5' to 3'	Note
mCherry	ATGGTGTCGAAGGGCGAG	CTTGTAGAGCTCGTCCATGC	
NAT	ATGGCGGCCGCCACTCTTGAC	TTATGGACAAGGCATACTCATATAAAG	
MAL-5F	TCCGTAGGTGAACCTGCGG		
MAL-4R		TCCTCCGCTTATTGATATGC	
ALID2078	TCCACGGTGCAGATCCTC		pACT1
ALID2081		CGTCCTCTCCTATGTCTG	pACT1
MfSAP1-ORF	ATGCAACTCAGCCTCAAGTTTGT	TTAGGCCTGGGTGGCGAAG	
MfSAP2-ORF	ATGCGTATCACCATCCCTCTTC	CTAGGCCTGCAAGGCAAAGC	
TD18003(S)-F	AGACTTGTCCAAATTGCTGGC		MFSAP1 5' Flanking Arm
TD18004(S)-R		TTGCATTGTGGATGGACGCT	MFSAP1 5' Flanking Arm
iN-TD18003-F	GTTTTCCCAGTCACGACGTTGTAAAACAGACTTGTCCAAATTGCTGGC		pGI3_MFSAP1-5’Flanking Arm
N-TD18004-R	CGAGGATCTGCACCGTGGA TTGCATTGTGGATGGACGCT		MFSAP1 5' Flanking Arm_pACT1
TD18038-F (S)-F	GGTACGAAGCCCACTCTTGT		MFSAP1 3' Flanking Arm
TD18046-R (S)-R		AGGTGACTATCCGTCCACGA	MFSAP1 3' Flanking Arm
TD18040-F	CTGGGTCAGACATAGGAGAGGACGGGTACGAAGCCCACTCTTGT		tACT1_MFSAP1 3' Flanking Arm
TD18047-R	CAGCTATGACATGATTACGAATTCTTAATTAGGTGACTATCCGTCCACGA		MFSAP1 3' Flanking Arm_pGI3

Table S7. List of qPCR primers used in murine colonization study.

Primer	Sequence 5' to 3'
Actb fwd	CCCTGAAGTACCCCATTGAAC
Actb rev	CTTTTCACGGTTGGCCTTAG
Cxcl1 fwd	CCGCTCGCTTCTCTGTG
Cxcl1 rev	GCAGCTCATTGGCGATAG
Cxcl2 fwd	AGTGAACTGCGCTGTCAATGC
Cxcl2 rev	GCAAACTTTTTGACCGCCCT
Cxcl5 fwd	GAAAGCTAAGCGGAATGCAC
Cxcl5 rev	GGGACAATGGTTTCCCTTTT
Il1b fwd	CAACCAACAAGTGATATTCTCCATG
Il1b rev	GATCCACACTCTCCAGCTGCA
Il6 fwd	GAGGATACCACTCCCAACAGACC
Il6 rev	AAGTGCATCATCGTTGTTCATACA
Il17 fwd	GCTCCAGAAGGCCCTCAGA
Il17 rev	AGCTTTCCCTCCGCATTGA
Defb3 fwd	GTC TCC ACC TGC AGC TTT TAG
Defb rev	ACT GCC AAT CTG ACG AGT GTT
Csf3 fwd	CTT AAG TCC CTG GAG CAA GTG
Csf3 rev	GTG GCC CAG CAA CAC CAG
Tnf fwd	CATCTTCTCAAAATTCGAGTGACAA
Tnf rev	TGGGAGTAGACAAGGTACAACCC

Software S1. Supplementary Analysis Code for targeted RNA-Seq barcoded sequenced reads.

SI References

1. V. Mandujano-González, L. Villa-Tanaca, M. A. Anducho-Reyes, Y. Mercado-Flores, Secreted fungal aspartic proteases: A review. *Rev. Iberoam. Micol.* **33**, 76–82 (2016).

2. G. Ianiri, A. F. Averette, J. M. Kingsbury, J. Heitman, A. Idnurm, Gene Function Analysis in the Ubiquitous Human Commensal and Pathogen *Malassezia* Genus. *MBio* **7**, 1–13 (2016).

3. W. Liao, *et al.*, Adiponectin induces interleukin-6 production and activates STAT3 in adult mouse cardiac fibroblasts. *Biol. Cell* **101**, 263–272 (2009).

4. L. Overbergh, *et al.*, The Use of Real-Time Reverse Transcriptase PCR for the Quantification of Cytokine Gene Expression. *J. Biomol. Tech.* **14**, 33 (2003).
